# Cytotoxic Meroterpenoids with Rare Skeletons from *Psidium guajava* Cultivated in Temperate Zone

**DOI:** 10.1038/srep32748

**Published:** 2016-09-02

**Authors:** Xu-Jie Qin, Huan Yan, Wei Ni, Mu-Yuan Yu, Afsar Khan, Hui Liu, Hong-Xia Zhang, Li He, Xiao-Jiang Hao, Ying-Tong Di, Hai-Yang Liu

**Affiliations:** 1 State Key Laboratory of Phytochemistry and Plant Resources in West China, Kunming Institute of Botany, Chinese Academy of Sciences, Kunming 650201, P. R. China; 2 University of Chinese Academy of Sciences, Beijing 100049, P. R. China; 3 Department of Chemistry, COMSATS Institute of Information Technology, Abbottabad-22060, Pakistan; 4 Department of Dermatology, The First Affiliated Hospital of Kunming Medical University, Kunming 650032, P. R. China

## Abstract

Three new meroterpenoids, guajavadials A–C (**1**–**3**), were isolated from *Psidium guajava* cultivated in temperate zone. Their structures were established by extensive spectroscopic evidence and electronic circular dichroism (ECD) calculations. Guajavadial A (**1**) represents a novel skeleton of the 3,5-diformylbenzyl phloroglucinol-coupled monoterpenoid, while guajavadials B (**2**) and C (**3**) are new adducts of the 3,5-diformylbenzyl phloroglucinol and a sesquiterpene with different coupling models. The plausible biosynthetic pathways as well as antimicrobial and cytotoxic activities of these meroterpenoids are also discussed. All these isolates exhibited moderate cytotoxicities against five human cancer cell lines, with **3** being most effective with an IC_50_ value of 3.54 *μ*M toward SMMC-7721 cell lines.

Meroterpenoids are an important class of hybrid natural products partially derived from terpenoid pathways, which have been found in marine organisms, fungi, and plants over the past half century^1^,^2^,^3^,^4^,^5^. Arising from their structural diversity, meroterpenoids exhibit various bioactivities, such as anticancer^6^, anti-inflammatory^7^, anti-biotic^8^, and antifibrotic^9^ activities. In recent years, the fascinating chemical structures and interesting biological activities of these compounds family have attracted great interest from the synthetic and pharmacological communities^10^,^11^,^12^.

*Psidium guajava* (family Myrtaceae), an arbor native to South America, is popularly cultivated in the subtropic and tropic regions of China^13^. Its leaves have been used as a folk medicine for the treatment of diarrhea and diabetes in China^14^. Previous investigations of the plant collected from Vietnam and Subtropic China have revealed that this plant is an abundant source of meroterpenoids^15^,^16^,^17^. Considering the fact that the plant secondary metabolites could be influenced significantly by the ecological environment, we examined the leaves of *P. guajava* collected from Panzhihua city of Sichuan province, China. In the current study, guajavadials A–C (**1**–**3**), three new meroterpenoids with rare skeletons were identified (Fig. 1). More importantly, all the isolates showed cytotoxicities against five human cancer cell lines. This paper describes the isolation, structural elucidation, plausible biosynthetic pathway, and bioactivity evaluation of **1**–**3**.

## Results and Discussion

Guajavadial A (**1**) was isolated as a colorless oil. The molecular formula of C_25_H_26_O_5_ was assigned by its positive HRESIMS at *m*/*z* 429.1672 [M+Na]^+^ (calcd for C_25_H_26_O_5_Na, 429.1678) with 13 degrees of unsaturation. The UV spectrum of **1** showed the absorption maxima at 283 and 372 nm. The IR spectrum showed characteristic absorption bands due to hydroxy (3439 cm^−1^), aldehyde (2930 cm^−1^), and aromatic rings (1633 and 1442 cm^−1^). The ^1^H NMR spectrum (Table 1) displayed the signals for two hydroxy groups (*δ*_H_ 13.64 and 13.25, each s), two aldehyde groups (*δ*_H_ 10.18 and 10.16, each s), five continuous aromatic protons (*δ*_H_ 7.26 × 2, t, *J* = 7.4 Hz; 7.19, t, *J* = 7.2 Hz; and 7.07 × 2, d, *J* = 7.5 Hz), and three methyls (*δ*_H_ 0.81, d, *J* = 6.8 Hz; 0.87, d, *J* = 6.8 Hz; and 1.03, s). The ^13^C NMR (Table 1) and HSQC spectra demonstrated 25 carbon signals, including two aldehyde groups, nine quaternary carbons, nine methines, two methylenes, and three methyls. Further analysis of these data suggested the characteristic signals for a 3,5-diformylbenzyl phloroglucinol (*δ*_C_ 192.2, 191.5, 169.8, 168.7, 163.3, 103.7 × 2, and 99.5), a monosubstituted aromatic ring (*δ*_C_ 142.5, 128.5 × 2, 127.2 × 2, and 126.5) and a methine at *δ*_C_ 35.1. The aforementioned data indicated **1** should be a phloroglucinol-coupled derivative.

**Figure 2:**
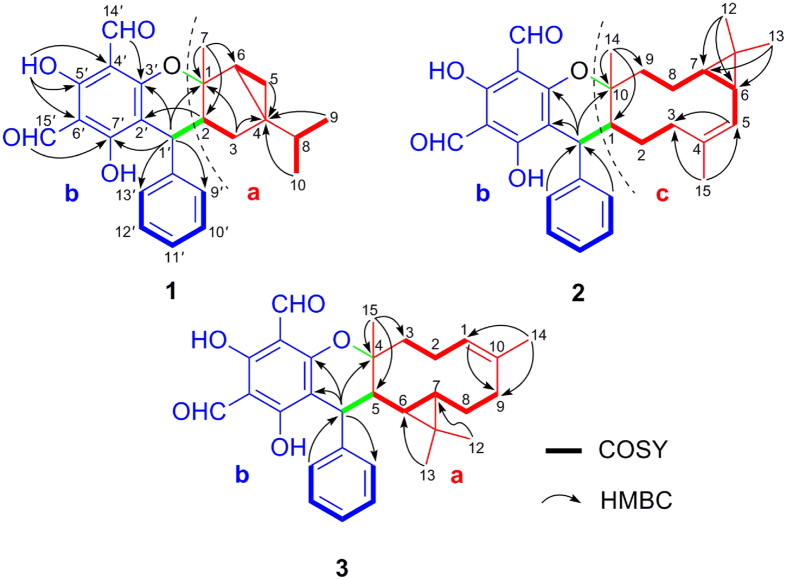
Key ^1^H–^1^H COSY and HMBC correlations of 1–3.

The gross structure of **1** was subsequently established by 2D NMR analysis (Fig. 2). The ^1^H–^1^H COSY spectrum revealed the presence of four spin systems (H-1′–H-2–H-3, H-5–H-6, H-9–H-8–H-10, and H-9′~H-13′). The HMBC correlations from Me-9/Me-10 to C-4, from Me-7 to C-2/C-6, from H-3a to C-1, and from H_2_-3/H-5a to C-4 indicated the presence of a monoterpenoid moiety “**a**” with a 3/5 bicyclic system and an isopropyl group at C-4. Moreover, the HMBC correlations from H-2 to C-2′ and from H-1′ to C-7′/C-9′, combined with the downfield shift at *δ*_C_ 163.3 (C-3′) and an oxygenated quaternary carbon at *δ*_C_ 89.8 (C-1) indicated the construction of the two structural fragments “**a**” and “**b**” via an ether linkage between C-1 and C-3′. With the aid of extensive analysis of its 2D NMR spectra, the assignments of ^1^H and ^13^C NMR data of **1** were achieved.

**Figure 3:**
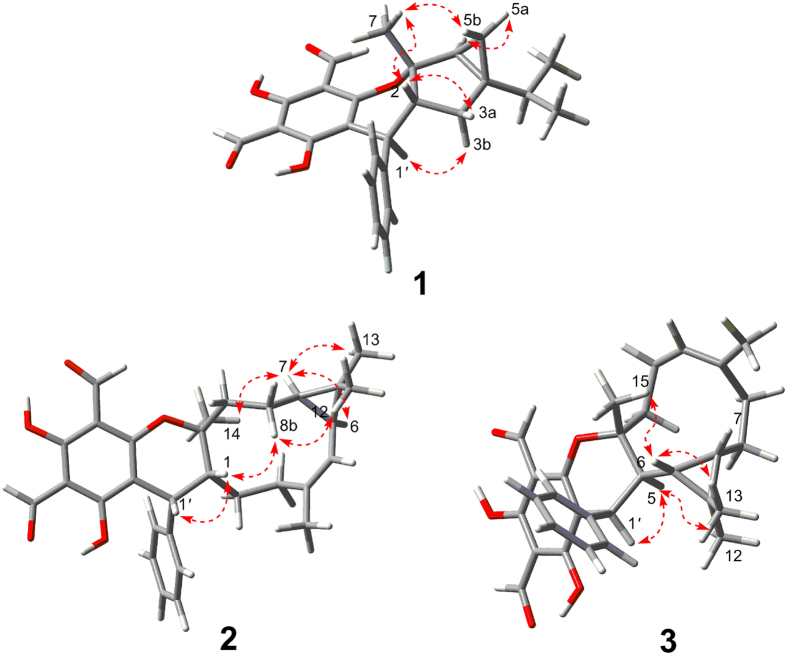
Key NOESY correlations of 1–3.

**Figure 4:**
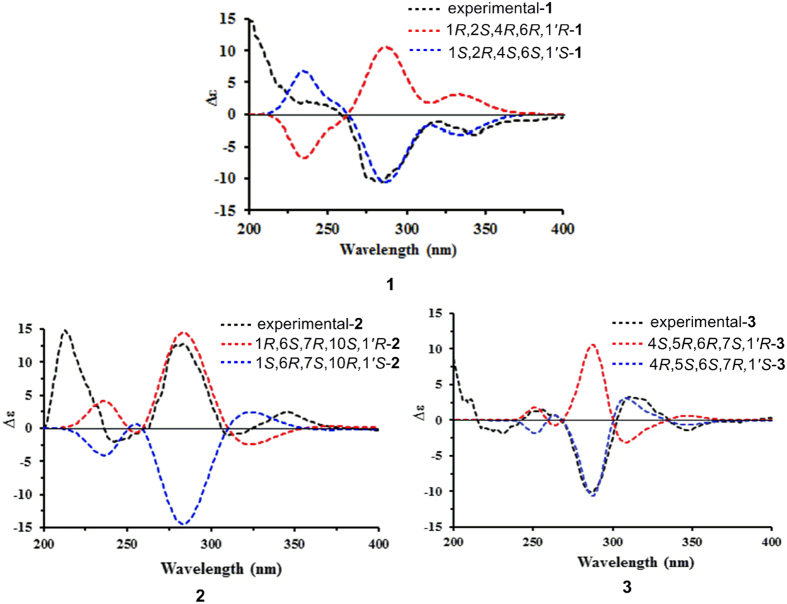
Calculated and experimental CD spectra of 1–3.

The relative configuration of **1** was determined by the analysis of NOESY spectrum (Fig. 3). NOE correlation between Me-7 and H-2 indicated that Me-7 and H-2 were *cis*-fused with the dihydropyran ring. In addition, diagnostic NOE correlations between Me-7 and H-5b, between H-2 and H-3a, between H-3b and H-1′, as well as between H-5a and H-6 suggested that these protons were placed on the same side, respectively. Unfortunately, many attempts to prepare a single crystal of this molecule have not been successful so far. Owing to indispensable methods for the stereochemical characterization of natural products^18^, the quantum mechanical calculations was performed to determine the absolute configuration of **1**. As illustrated in Fig. 4, the well matched result between the calculated ECD curve and the measured one led to the assignment of its absolute configuration as 1*S*,2*R*,4*S*,6*S*,1′*S*. Based on the above evidence, the structure of **1** was established as shown.

Guajavadial B (**2**) was obtained as a white amorphous powder. Its molecular formula, C_30_H_34_O_5_, was deduced from the positive HRESIMS at *m*/*z* 475.2477 [M+H]^+^ (calcd for C_30_H_35_O_5_, 475.2485), suggesting 14 degrees of unsaturation. Detailed comparison of its ^1^H and ^13^C NMR data with those of **1** suggested that they both possessed the same structural moiety of 3,5-diformylbenzyl phloroglucinol “**b**”. Two spin systems of H-1′–H-1~H-3 and H-5~H-9 were revealed by the ^1^H–^1^H COSY spectrum, which in combination with the HMBC correlations from Me-15 to C-3/C-5 and from Me-14 to C-1/C-9/C-10, indicated the existence of a 10-membered ring in **2**. Likewise, the HMBC correlations of Me-12/Me-13 with C-6/C-7 suggested the presence of a cyclopropane ring between C-6 and C-7. Thus, the sesquiterpene unit “**c**” with a 3/10 continuous carbocyclic system was established. Besides, the downfield shifts at *δ*_C_ 164.4 (C-3′) and 88.3 (C-10) suggested an oxygen atom bridging C-10 and C-3′ to form a dihydropyran ring, which was also consistent with the HMBC correlations from H-1′ to C-10/C-2′/C-3′ and the unsaturation degrees obtained from the HRMS. The NOE correlations between H-7 and Me-14/Me-13, between Me-12 and H-8b, as well as between H-8b and H-1 settled the relative configuration of **2**. Furthermore, the coupling constant between H-1′ and H-1 (5.9 Hz) indicated those protons should be placed at the same side, which was further confirmed by NOE correlations of these two protons (Fig. 3). Finally, a good agreement between the calculated ECD and experimental CD spectra suggested the absolute configuration of **2** as 1*R*,6*S*,7*R*,10*S*,1′*R*.

Guajavadial C (**3**) was shown to have the same molecular formula as **2** by its HRESIMS. The characteristic NMR signals indicated that **3** was also a sesquiterpene-based meroterpenoid similar to **2** (Table 2). Three structural fragments (C-1–C-3, C-1′–C-5–C-9 and C-9′–C-13′) were also secured by the ^1^H–^1^H COSY spectrum, and detailed analysis of 2D NMR spectra (Fig. 2) revealed that its sesquiterpene unit “**c**” was identical to that of **2**. However, the key HMBC correlations from Me-15 to C-3/C-4/C-5, together with the downfield shift at *δ*_C_ 91.7 (C-4) defined the connection of fragments “**c**” and “**b**”. Thus, compounds **2** and **3** are the regio-isomers. The NOE correlations between H-6 and Me-12/Me-15, as well as between Me-13 and H-5/H-1′ assigned the relative configuration of **3**. The absolute configuration of **3** was determined as 4*R*,5*S*,6*S*,7*R*,1′*S* by comparing its experimental CD spectrum with the calculated one (Fig. 3).

**Figure 5:**
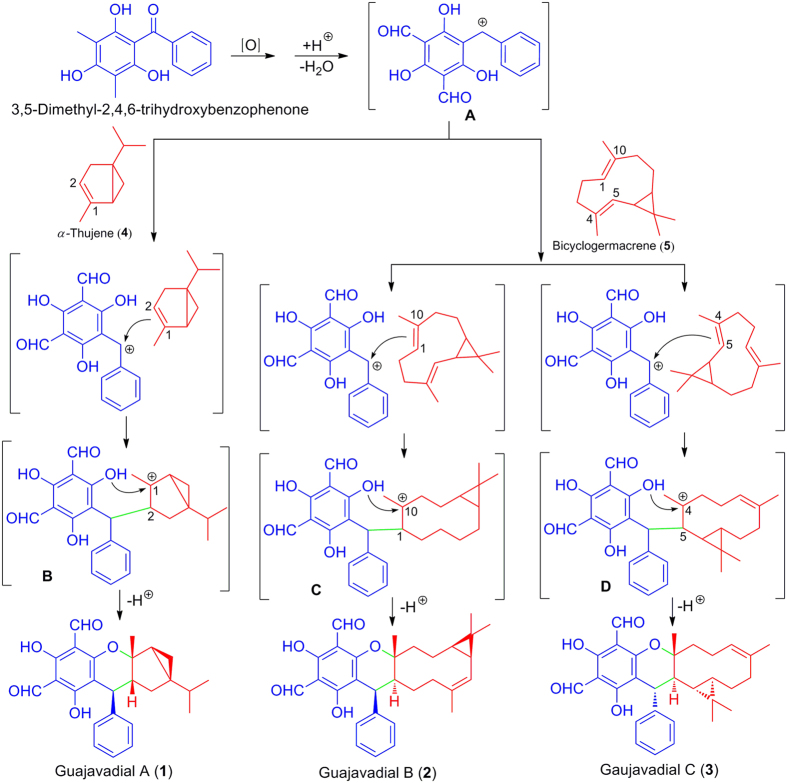
Plausible Biosynthetic Pathways for 1–3.

Biosynthetically, **1**–**3** are the new adducts derived from a phloroglucinol precursor (3,5-dimethylbenzyl-2,4,6-trihydroxybenzophenon)^19^ and two terpene precursors (**4** and **5**) via hetero-Diels-Alder reactions. Fortunately, two terpene precursors *α*-thujene (**4**) and bicyclogermacrene (**5**) were identified by GC-MS analysis in the essential oil from the leaves *P. guajava* collected from temperate zone (see the Supplementary Information). Briefly, 3,5-dimethyl-2,4,6-trihydroxybenzophenon could be oxidized followed by losing an H_2_O under acidic condition and then generated a carbocation **A**. As a cationic initiator, the carbocation **A** would couple with different terpene precursors, *α*-thujene (**4**) and bicyclogermacrene (**5**), respectively, to give carbocations **B**–**D**. Further rearrangement of intermediates **B**–**D** could establish different meroterpenoids skeletons to finally generate guajavadials A–C (**1**–**3**). The plausible biosynthetic pathways of **1**–**3** are shown in Fig. 5.

The antimicrobial activities **1**–**3** toward five microbial strains (*P. aeruginosa, S. aureus, E. coli, M. canis*, and *M. gypseum*) were evaluated as described previously^20^. Unfortunately, none of them exhibited obvious antimicrobial activities against three bacteria and two fungi at the concentrations of 100 *μ*g/mL and 250 *μ*g/mL, respectively. In addition, the inhibitory effects of the isolates against five human cancer cell lines (HL-60, A-549, SMMC-7721, MCF-7, and SW480) were also tested by MTT method^21^. All these meroterpenoids showed moderate cytotoxic activities ranging from 22.28 to 3.38 *μ*M (Table 3), with **3** being the most effective with an IC_50_ value of 3.54 *μ*M toward SMMC-7721 cell lines compared to positive control cisplatin (IC_50_ 19.82 *μ*M). These result suggest that *P. guajava* leaves could provide a source of potential therapeutic compounds for both the prevention and treatment of cancer^22^.

In conclusion, a phytochemistry investigation on the leaves of *Psidium guajava* cultivated in temperate zone led to the discovery of a novel meroterpenoid featuring an unprecedented skeleton of the 3,5-diformylbenzyl phloroglucinol-coupled monoterpenoid, guajavadial A (**1**), together with two new ones possessed the 3,5-diformylbenzyl phloroglucinol and a sesquiterpene with different coupling models, guajavadials B (**2**) and C (**3**). This finding of **1**–**3** could provide evidence that the plant secondary metabolites can be influenced by the ecological environment. Besides, these isolates might provide challenging natural products for organic synthesis and enrich the diversity of meroterpenoids via hetero-Diels-Alder reactions. All these meroterpenoids did not display antimicrobial activity but exhibited cytotoxicity effects against five human cancer cell lines.

## Methods

### General Experimental Procedures

Optical rotations were measured on a JACSO P-1020 polarimeter. UV spectra were taken on a Shimadzu UV-2401PC spectrometer. IR spectra were determined on a Bruker FT-IR Tensor-27 infrared spectrophotometer with KBr disks. ECD spectra were recorded with an Applied Photophysics Chirascan Spectrometer in MeOH. 1D and 2D NMR spectra were recorded on Bruker DRX-600 spectrometers with the solvent signal as an internal standard. ESIMS and HRESIMS analyses were carried out on Waters Xevo TQS and Agilent G6230 TOF mass spectrometers, respectively. Silica gel (200–300 mesh, Qingdao Marine Chemical Co., Ltd., China), RP-18 (40–63 *μ*m, Merck, Darmstadt, Germany) and Sephadex LH-20 (Pharmacia Uppsala, Sweden) were used for column chromatography. Semi-preparative HPLC was performed on an Agilent 1260 HPLC with a ZORBAX SB-C18 (9.4 × 250 mm) column. Fractions were monitored by TLC (GF254, Qingdao Marine Chemical Co., Ltd.). Compounds were visualized under UV254 light as well as spraying with 10% H_2_SO_4_ in EtOH solution (V/V), followed by heating.

### Plant Material

The leaves of *Psidium guajava* (Myrtaceae) were collected from Panzhihua city, Sichuan province, People’s Republic of China, in June 2015. The herbarium specimen was authenticated by Dr. Rong Li (Kunming Institute of Botany, Chinese Academy of Sciences). A voucher specimen (HY0024) was deposited in the State Key Laboratory of Phytochemistry and Plant Resources in West China, Kunming Institute of Botany, Chinese Academy of Sciences.

### Extraction and Isolation

The air-dried powdered leaves of *P. guajava* (5.0 kg) were percolated with 95% EtOH (45 L) at room temperature (48 h × 3). The EtOH residue (1.2 kg) was suspended in H_2_O and then partitioned with ethyl acetate (EtOAc). The EtOAc extract (500 g) was subjected to silica gel column eluted with a gradient mixture of PE (petroleum ether)-EtOAc (100:1 → 0:100) to give six main fractions (A–F). Fraction B (31.2 g) was subsequently subjected to Sephadex LH-20 column eluted with CHCl_3_-MeOH (3:2) to obtain three fractions (B.1–B.3). Fraction B.2 (9.2 g) was chromatographed over RP-18 column eluted with MeCN-H_2_O (80:20 → 100:0) to yield five fractions (B.2.1–B.2.5). Fraction B.2.3 (150 mg) was further purified by semi-preparative HPLC (flow rate: 4 mL/min; detection wavelength: 275 nm; mobile phase: MeCN-0.01% TFA, 88:12, V/V) to afford compounds **1** (25 mg), **2** (13 mg), and **3** (10 mg).

### GC-MS Analysis of 4 and 5

The air-dried and powdered leaves of *Psidium guajava* (100 g) were hydrodistilled for 2 h using a Clevenger apparatus to obtain the essential oil (0.5 mL). An Agilent 7890A gas chromatograph was used with helium as a carrier gas at a flow rate of 2.2 mL/min, 20:1 split injection (injector temperature 250 °C, injection volume 1.0 *μ*L), using an Agilent HP-5 MS column (50 m × 0.32 mm, 0.52 μm film thickness) and a temperature program from 40 °C to 250 °C at a rate of 2 °C/min, followed by 5 °C/min until 250 °C (10 min hold). The coupled mass spectrometer was an Agilent model 5975C system, transfer line temperature 280 °C, source temperature 230 °C, ionization potential 70 eV, and a scan range of 30–400 atomic mass units (amu). Compounds were identified by comparison of experimental spectra with the reference spectra in NIST14 data base. About twenty volatile components, including *α*-thujene (**4**, *t*_R_: 5.981 min) and bicyclogermacrene (**5**, *t*_R_: 27.218 min) were identified in this research.

### ECD Calculation of 1–3

The theoretical calculations of **1**–**3** were performed using Gaussian 09^23^. Briefly, the 3D structures of **1**–**3** were first established according to the NOESY spectra. Their 3D structures were then performed in the SYBYL 8.1 program by using MMFF94s molecular force field. All the obtained conformers were optimized using DFT at the B3LYP/6-31+G(d) level in gas phase. Further optimization at the B3LYP/6-311G(2d,p) level led the dihedral angles to be got. The optimized conformations were used for the ECD calculations using time dependent Density Functional Theory (TDDFT). The ECD spectra were produced by SpecDis 1.6 software^24^. The calculated ECD spectra of **1**–**3** were subsequently compared with the experimental ones.

The ECD spectra were simulated by overlapping Gaussian functions for each transition according to:


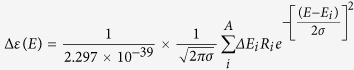


where *σ* represents the width of the band at 1/*e* height, and Δ*E*_*i*_ and *R*_*i*_ are the excitation energies and rotational strengths for transition *i*, respectively. *σ* = 0.30 eV and *R*_vel_ were used in this work.

## Antimicrobial Assay

### Microorganisms and culture media

The microorganisms used included three bacterial strains: *Pseudomonas aeruginosa* (ATCC 27853), *Staphylococcus aureus* (ATCC 25922) and *Escherichia coli* (ATCC 11775); two dermatophytes: *Microsporium canis* (CBS 113480) and *Microsporium gypseum* (E1420). They were obtained from the American Type Culture Collection (ATCC) and “Centre Pasteur” of Yaounde Cameroon, respectively. Nutrient agar (NA, Conda) and Sabouraud dextrose agar (SDA, Conda) media were used for culturing bacteria and fungi, respectively, while Mueller Hinton broth (MHB, Conda), and Sabouraud dextrose broth (SDB, Conda) were used for the determination of minimum inhibitory concentrations (MIC).

### Preparation of microbial inocula

The inocula of bacteria were prepared from 24 h old agar cultures. The absorbance was read at 600 and 530 nm, respectively, and adjusted with sterile physiological solution to an absorbance of 0.10 (0.5 McFarland standards). These solutions corresponded to 10^8^ CFU/mL for bacteria. From the prepared bacterial solutions, other dilutions were prepared to give a final concentration of 10^6^ CFU/mL. Conidia suspensions of dermatophyte species were prepared from 10 days old cultures. The number of conidia was determined using a spectrophotometer and adjusted with 0.9% sterile saline solution to an absorbance of 0.600 at 450 nm, corresponding to a final concentration of 4 × 10^3^ spores/mL.

### Determination of minimum inhibitory concentration (MIC)

The MICs of the isolated compounds were determined in 96-well micro-titre plates by the broth microdilution method. The 96-well plates were prepared by dispensing into each well 100 *μ*L of Mueller Hinton broth for bacteria and Sabouraud dextrose broth for fungi. The samples were initially prepared in 10% DMSO in broth medium at 400 *μ*g/mL for the tested compounds or 50 *μ*g/mL for the reference antibiotics. A volume of 100 *μ*L of each test sample was added into the first wells of the micro-titre plate (whose wells were previously loaded with 100 *μ*L of broth medium). Serial two-fold dilutions of the test samples were made and 100 *μ*L of the inocula were added into the respective wells. This gave final concentration ranges of 100 to 0.781 *μ*g/mL for the compounds and 12.5 to 0.097 *μ*g/mL for reference substances. The plates were sealed with parafilm, then agitated with a plate shaker to mix their contents and incubated at 35 °C for 24 h for bacteria and at 28 °C for 5 days for dermatophytes.

For bacteria, MICs were determined upon addition of 50 *μ*L (0.2 mg/mL) *p*-iodonitrotetrazolium chloride (INT, Sigma-Aldrich). Viable bacteria reduced the yellow dye to a pink color. For dermatophytes, MICs were determined by visualizing the turbidity of the wells. The MIC was defined as the lowest concentration where no color or turbidity change was observed, indicating no visible growth of microorganism. All the tests were performed in triplicates. Gentamycin for bacteria and griseofulvin for dermatophytes were used as positives controls, respectively.

## Cell Growth Inhibition Assay

### The following human cancer cell lines were used

SW-480, SMMC-7721, HL-60, MCF-7, and A-549. All the cells were cultured in RPMI-1640 or DMEM medium (Hyclone, Logan, UT), supplemented with 10% fetal bovine serum (Hyclone) at 37 °C in a humidified atmosphere with 5% CO_2_. Cell viability was assessed by conducting colorimetric measurements of the amount of insoluble formazan formed in living cells based on the reduction of 3-(4,5-dimethylthiazol-2-yl)-2, 5-diphenyltetrazolium bromide (MTT) (Sigma, St. Louis, MO). Briefly, 100 μL of adherent cells were seeded into each well of a 96-well cell culture plate and allowed to adhere for 12 h before drug addition, while suspended cells were seeded just before drug addition, both with an initial density of 1 × 10^5^ cells/mL in 100 *μ*L of medium. Each cell line was exposed to the test compound at various concentrations in triplicate for 48 h, with cisplatin as positive control. After the incubation, MTT (100 *μ*g) was added to each well, and the incubation continued for 4 h at 37 °C. The cells were lysed with 100 *μ*L of 20% SDS-50% DMF after removal of 100 *μ*L of medium. The absorbance of the lysate was measured at 595 nm in a 96-well Microtiter plate reader (Bio-Rad 680).

Guajavadial A (**1**): colorless oil; [*α*]_D_^22^ −70.8 (*c* 0.13, MeOH); UV (MeOH) *λ*_max_ (log *ε*) 283 (4.61), 372 (3.52) nm; IR (KBr) *ν*_max_ 3439, 2958, 2930, 2871, 1633, 1442, 1383, 1307, 1203, 1179, 1082, 835 cm^−1^; CD (MeOH) *λ*_max_ (*Δε*) 285 (−10.76), 344 (−3.18) nm; ^1^H (CDCl_3_, 600 MHz) and ^13^C (CDCl_3_, 150 MHz) NMR data, see Table 1; ESIMS *m*/*z* 429 [M + Na]^+^; HRESIMS *m*/*z* 429.1672 [M + Na]^+^ (calcd for C_25_H_26_O_5_Na, 429.1678).

Guajavadial B (**2**): white amorphous powder; [*α*]_D_^24^ +109.3 (*c* 0.10, MeOH); UV (MeOH) *λ*_max_ (log *ε*) 289 (4.60), 377 (3.58) nm; IR (KBr) *ν*_max_ 3441, 2953, 2922, 1632, 1444, 1385, 1302, 1219, 1182, 1092, 842 cm^−1^; CD (MeOH) *λ*_max_ (*Δε*) 242 (−2.1), 283 (+13.3), 310 (−1.1), 346 (+2.6) nm; ^1^H (CDCl_3_, 600 MHz) and ^13^C (CDCl_3_, 150 MHz) NMR data, see Table 2; ESIMS *m*/*z* 475 [M + H]^+^; HRESIMS *m*/*z* 475.2477 [M + H]^+^ (calcd for C_30_H_35_O_5_, 475.2485).

Guajavadial C (**3**): white amorphous powder; [*α*] _D_^22^ –44.7 (*c* 0.12, MeOH); UV (MeOH) *λ*_max_ (log *ε*) 295 (4.43), 374 (3.64) nm; IR (KBr) *ν*_max_ 3441, 2961, 2922, 2858, 1637, 1444, 1385, 1305, 1218, 1176, 1053, 841 cm^−1^; CD (MeOH) *λ*_max_ (*Δε*) 256 (+1.8), 287 (−12.1), 311 (+3.9), 346 (−1.8) nm; ^1^H (CDCl_3_, 600 MHz) and ^13^C (CDCl_3_, 150 MHz) NMR data, see Table 2; ESIMS *m*/*z* 475 [M + H]^+^; HRESIMS *m*/*z* 475.2485 [M + H]^+^ (calcd for C_30_H_35_O_5_, 475.2485).

## Additional Information

**How to cite this article**: Qin, X.-J. *et al*. Cytotoxic Meroterpenoids with Rare Skeletons from *Psidium guajava* Cultivated in Temperate Zone. *Sci. Rep.* **6**, 32748; doi: 10.1038/srep32748 (2016).

## Supplementary Material

Supplementary Information

## Figures and Tables

**Figure 1:**
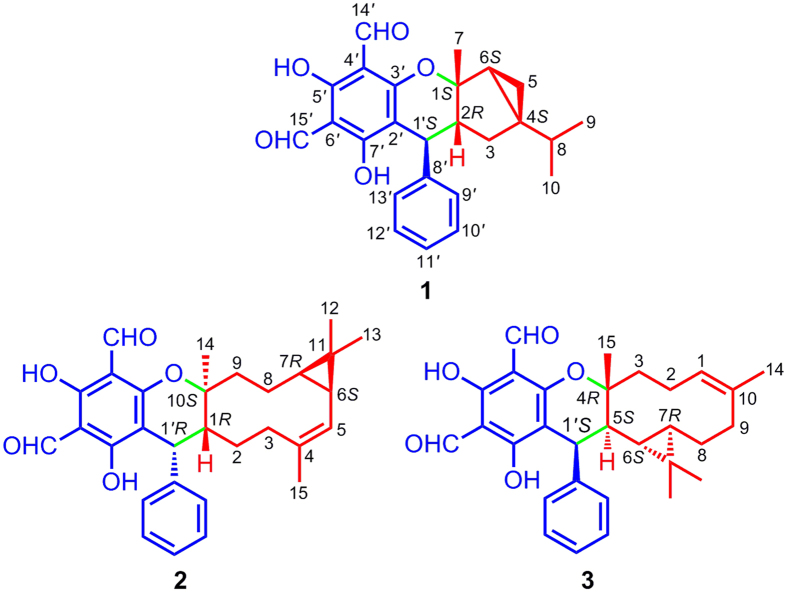
Chemical structures of 1–3.

**Table 1:** NMR Data of 1 in CDCl_3_ (*δ* in ppm, *J* in Hz).

No.	*δ*_H_	*δ*_C_	No.	*δ*_H_	*δ*_C_
1	—	89.8 (s)	1*'*	4.20 (brs)	35.1 (d)
2	2.23 (t, 9.2)	44.0 (d)	2*'*	—	99.5 (s)
3	a 2.03 (dd, 12.6, 7.6)	34.1 (t)	3*'*	—	163.3 (s)
	b 1.59 (t, 11.9)		4′	—	103.7 (s)
4	—	32.5 (s)	5′	—	168.7 (s)
5	a 0.57 (t, 6.8)	15.0 (t)	6′	—	103.7 (s)
	b 0.49 (m)		7′	—	169.8 (s)
6	1.38 (dd, 8.1, 2.6)	35.2 (d)	8′	—	142.5 (s)
7	1.03 (s)	23.4 (q)	9′	7.07 (d, 7.5)	127.2 (d)
8	1.31 (m)	32.1 (d)	10′	7.26 (t, 7.4)	128.5 (d)
9	0.81 (d, 6.8)	19.5 (q)	11*'*	7.19 (t, 7.2)	126.5 (d)
10	0.87 (d, 6.8)	19.9 (q)	12′	7.26 (t, 7.4)	128.5 (d)
5′-OH	13.64 (s)		13′	7.07 (d, 7.5)	127.2 (d)
7′-OH	13.25 (s)		14′	10.18 (s)	192.2 (d)
			15′	10.16 (s)	191.5 (d)

**Table 2:** NMR Data of 2 and 3 (*δ* in ppm, *J* in Hz).

No.	2^a^		3^a^	
	*δ*_H_	*δ*_C_	*δ*_H_	*δ*_C_
1	2.27 (brt, 5.3)	38.7 (d)	5.19 (brd, 11.5)	123.4 (d)
2	a 1.99	24.4 (t)	a 2.33 (qd, 13.3, 4.1)	23.6 (t)
2	b 0.89 (brt, 13.3)		b 1.99 (brd, 12.7)	
3	a 2.23 (dd, 12.5, 5.1)	38.8 (t)	a 1.84 (dt, 13.5, 4.9)	43.2 (t)
3	b 2.00 (brt, 13.0)		b 1.58 (td, 13.2, 4.5)	
4	—	136.5 (s)	—	91.7 (s)
5	4.92 (d, 8.9)	121.7 (d)	2.71 (brd, 8.0)	49.1 (d)
6	1.21 (t, 8.8)	24.8 (d)	0.38 (brd, 9.2)	29.2 (d)
7	0.70 (m)	27.6 (d)	0.39	28.6 (d)
8	a 1.58 (m)	19.2 (t)	a 1.91 (brd, 14.3)	25.4 (t)
8	b 0.75 (q, 11.4)		b 1.37 (m)	
9	a 1.82 (dt, 14.4, 9.2)	39.3 (t)	a 2.46 (brd, 14.5)	34.9 (t)
9	b 1.17 (dd, 14.4, 9.2)		b 1.64 (m)	
10	—	88.3 (s)	—	133.9 (s)
11	—	20.4 (s)	—	nd^b^
12	1.03 (s)	28.7 (q)	0.97 (s)	30.1 (q)
13	1.04 (s)	15.2 (q)	1.28 (s)	17.2 (q)
14	1.12 (s)	24.6 (q)	1.69 (s)	21.0 (q)
15	1.68 (s)	17.0 (q)	1.22 (s)	24.5 (q)
1′	4.17 (d, 5.9)	36.7 (d)	4.54 (brs)	38.0 (d)
2′	—	105.7 (s)	—	108.3 (s)
3′	—	164.4 (s)	—	163.0 (s)
4′	—	103.4 (s)	—	104.9 (s)
5′	—	167.9 (s)	—	167.6 (s)
6′	—	103.8 (s)	—	106.6 (s)
7′	—	168.7 (s)	—	168.1 (s)
8′	—	139.7 (s)	—	141.8 (s)
9′	7.15 (d, 7.5)	130.7 (d)	7.11 (d, 7.6)	127.5 (d)
10′	7.25 (t, 7.3)	127.7 (d)	7.26 (t, 7.5)	128.4 (d)
11′	7.21 (t, 7.1)	126.9 (d)	7.18 (t, 7.3)	126.2 (d)
12′	7.25 (t, 7.3)	127.7 (d)	7.26 (t, 7.5)	128.4 (d)
13′	7.15 (d, 7.5)	130.7 (d)	7.11 (d, 7.6)	127.5 (d)
14′	10.11 (s)	192.0 (d)	10.25 (s)	192.2 (d)
15′	10.09 (s)	191.5 (d)	10.04 (s)	192.0 (d)
5′-OH	13.55 (s)		13.21 (s)	
7′-OH	13.13 (s)		13.00 (s)	

^a^Overlapped signals are reported without designating multiplicity.

^b^Not detected.

**Table 3:** Cytotoxicities with IC_50_ values of 1–3 (*μ*M).

	HL-60	A-549	SMMC-7721	MCF-7	SW480
**1**	4.73	5.62	4.37	22.28	14.55
**2**	6.49	5.78	5.05	18.02	13.07
**3**	3.38	5.66	3.54	14.54	18.97
Cisplatin^a^	2.02	13.71	19.82	14.64	20.19

^a^Positive control.
