# Supplementary material for: Cytotoxic Meroterpenoids with Rare Skeletons from Psidium guajava Cultivated in Temperate Zone
Source: Sci Rep. 2016 Sep 2;6:32748. doi: 10.1038/srep32748 (PMC5009466; doi:10.1038/srep32748)
Supplement: Supplementary Information [file srep32748-s1.doc]

**Supplementary Information for**

**Cytotoxic Meroterpenoids with Rare Skeletons from *Psidium guajava* Cultivated in Temperate Zone**

Xu-Jie Qin, Huan Yan, Wei Ni, Mu-Yuan Yu, Afsar Khan, Hui Liu, Hong-Xia Zhang, Li He, Xiao-Jiang Hao, Ying-Tong Di, and Hai-Yang Liu

**Content**

**GC-MS analysis of 4 and 5**…………………………………………….….…….S2

**Computational data of 1–3**……………………………………………………S3–S8

**Original NMR, MS and CD spectra**……………………………………...…..S9–S20

**Figures S1–S6.** NMR spectra of guajavadial A (**1**)………………………..…...S8–S11

**Figure S7.** HRESIMS spectrum of guajavadial A (**1**) …………………..…..…....S12

**Figures S8.** CD spectrum of guajavadial A (**1**)…………………………..……….S12

**Figures S9–S14.** NMR spectra of guajavadial B (**2**)……………………….....S13–S15

**Figure S15.** HRESIMS spectrum of guajavadial B (**2**)……………………..……S16

**Figure S16.** CD spectrum of guajavadial B (**2**)……………………….…..…...…S16

**Figure S17–S22.** NMR spectra of guajavadial C (**3**)…………........................S17–S19

**Figure S23.** HRESIMS spectrum of guajavadial C (**3**)……………………..……S20

**Figure S24.** CD spectrum of guajavadial C (**3**)……………………….…..…...…S20

**GC-MS analysis of 4 and 5**

**A: Total ion chromatograms (TICs) of the essential oil of *Psidium guajava*.**


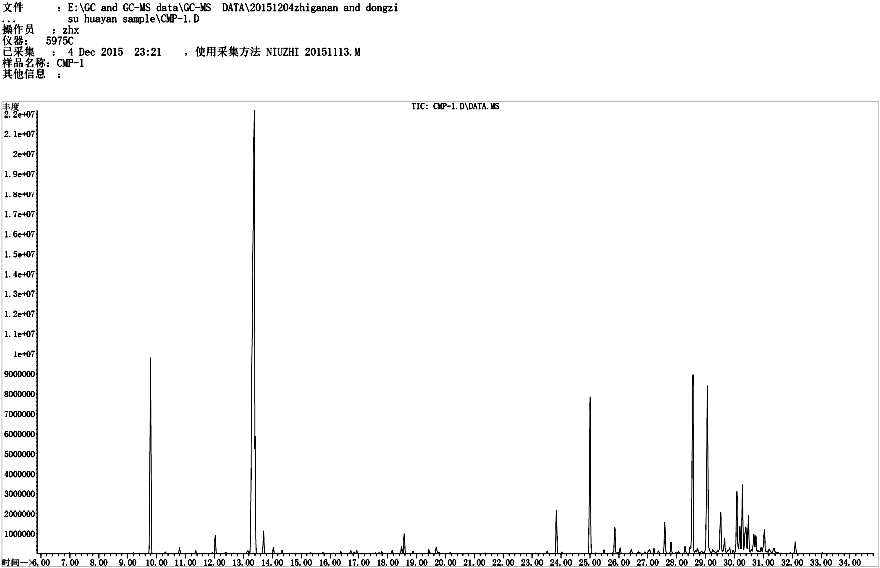


**B: The mass spectrum of *α*-thujene (4)**

*t*_R_: 9.581 min; C_10_H_16_; M. 98.7%

**C: The mass spectrum of bicyclogermacrene (5)**

*t*_R_: 27.218 min; C_15_H_46_; M. 98.4%

**Computational data of 1–3**

**Optimized structure of** 1*R*,2*S*,4*R*,6*R*,1*'R*-**1**


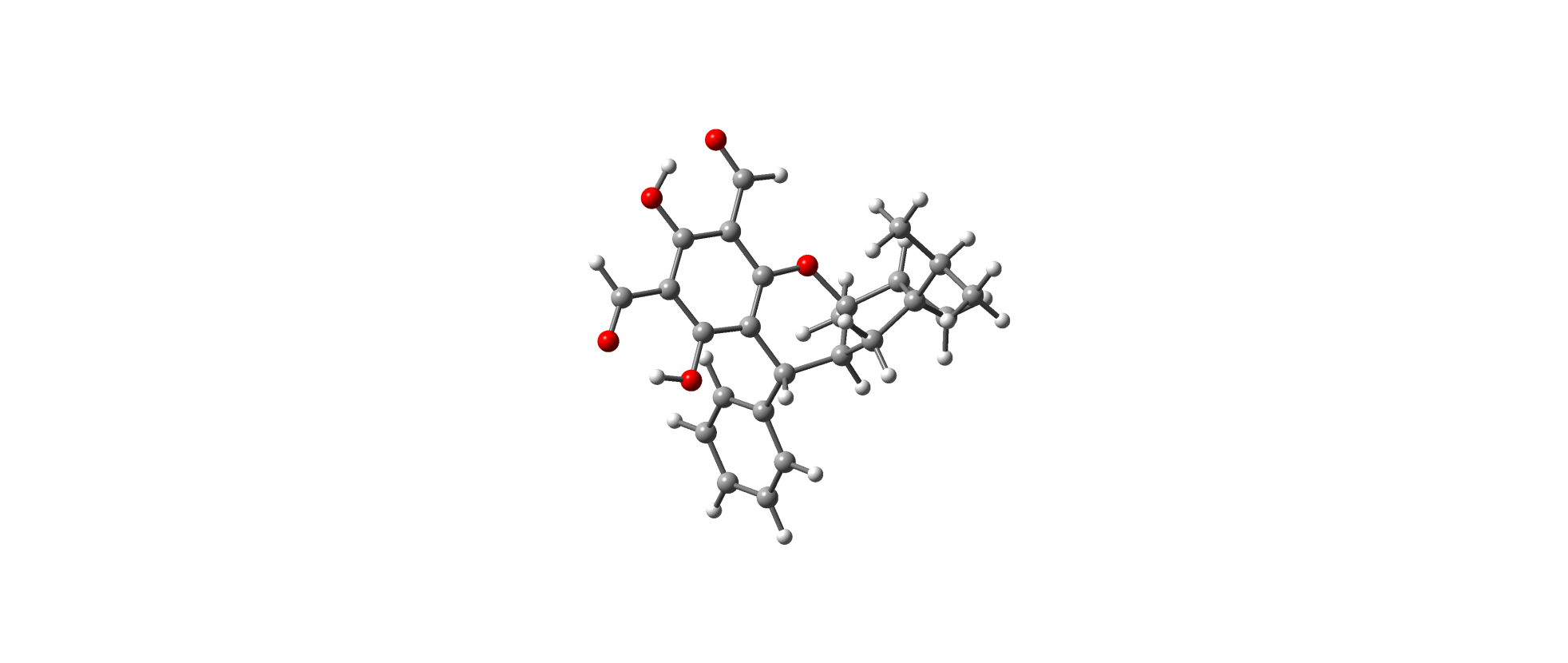


Standard orientation:

------------------------------------------------------------------------------------------------------

Center Atomic Atomic Coordinates (Angstroms)

Number Number Type X Y Z

------------------------------------------------------------------------------------------------------

1 6 0 -1.372169 -2.904975 0.375287

2 6 0 -2.137865 -2.290347 -0.615916

3 6 0 -1.864333 -0.962356 -0.978300

4 6 0 -0.832755 -0.232146 -0.355122

5 6 0 -0.062909 -0.857738 0.632326

6 6 0 -0.335080 -2.189724 0.996761

7 6 0 -0.569148 1.207722 -0.740913

8 6 0 0.892435 1.570283 -0.460296

9 6 0 1.420873 1.052101 0.896562

10 8 0 0.965475 -0.248101 1.286590

11 8 0 -2.582430 -0.322960 -1.958700

12 8 0 -1.659452 -4.205310 0.711154

13 6 0 -1.532891 2.233510 -0.140446

14 6 0 -1.653151 3.489949 -0.762644

15 6 0 -2.509230 4.468328 -0.253457

16 6 0 -3.263586 4.207961 0.886423

17 6 0 -3.163543 2.969468 1.514090

18 6 0 -2.306754 1.989538 1.005126

19 6 0 1.861985 0.963346 -1.514663

20 6 0 3.188471 0.782244 -0.794771

21 6 0 2.921259 0.918283 0.676294

22 6 0 3.746231 1.969407 -0.023082

23 6 0 4.084829 -0.332835 -1.330123

24 6 0 5.564409 -0.184891 -0.974584

25 6 0 3.589696 -1.716167 -0.890528

26 6 0 1.157101 2.004055 2.062660

27 1 0 1.030504 2.659550 -0.512973

28 6 0 -3.225290 -3.044712 -1.272726

29 8 0 -3.909748 -2.517274 -2.146961

30 6 0 0.489099 -2.838598 2.040286

31 8 0 0.265898 -4.000603 2.373821

32 1 0 -0.701478 1.276021 -1.831212

33 1 0 -3.284906 -0.931110 -2.286588

34 1 0 -1.034816 -4.497080 1.417583

35 1 0 -1.080364 3.712821 -1.660945

36 1 0 -2.590659 5.431068 -0.751115

37 1 0 -3.933417 4.966613 1.281794

38 1 0 -3.756441 2.762100 2.401278

39 1 0 -2.257305 1.036422 1.524547

40 1 0 1.973507 1.638434 -2.370449

41 1 0 1.492581 0.006174 -1.896027

42 1 0 3.420285 0.282226 1.394945

43 1 0 4.797799 2.020290 0.234263

44 1 0 3.323010 2.953368 -0.194431

45 1 0 4.022379 -0.294055 -2.425879

46 1 0 6.153498 -0.993008 -1.421956

47 1 0 5.966686 0.760273 -1.354151

48 1 0 5.724822 -0.215645 0.108434

49 1 0 4.191177 -2.507168 -1.351254

50 1 0 2.548285 -1.882023 -1.182445

51 1 0 3.651650 -1.838279 0.196060

52 1 0 0.101099 2.020794 2.340403

53 1 0 1.699507 1.674435 2.957109

54 1 0 1.479324 3.025727 1.837160

55 1 0 -3.402487 -4.085865 -0.957153

56 1 0 1.301741 -2.258327 2.506310 ---------------------------------------------------------------------------------------------------------

Optimized structure of 1*R*,6*S*,7*R*,10*S*,1*'R*-**2**


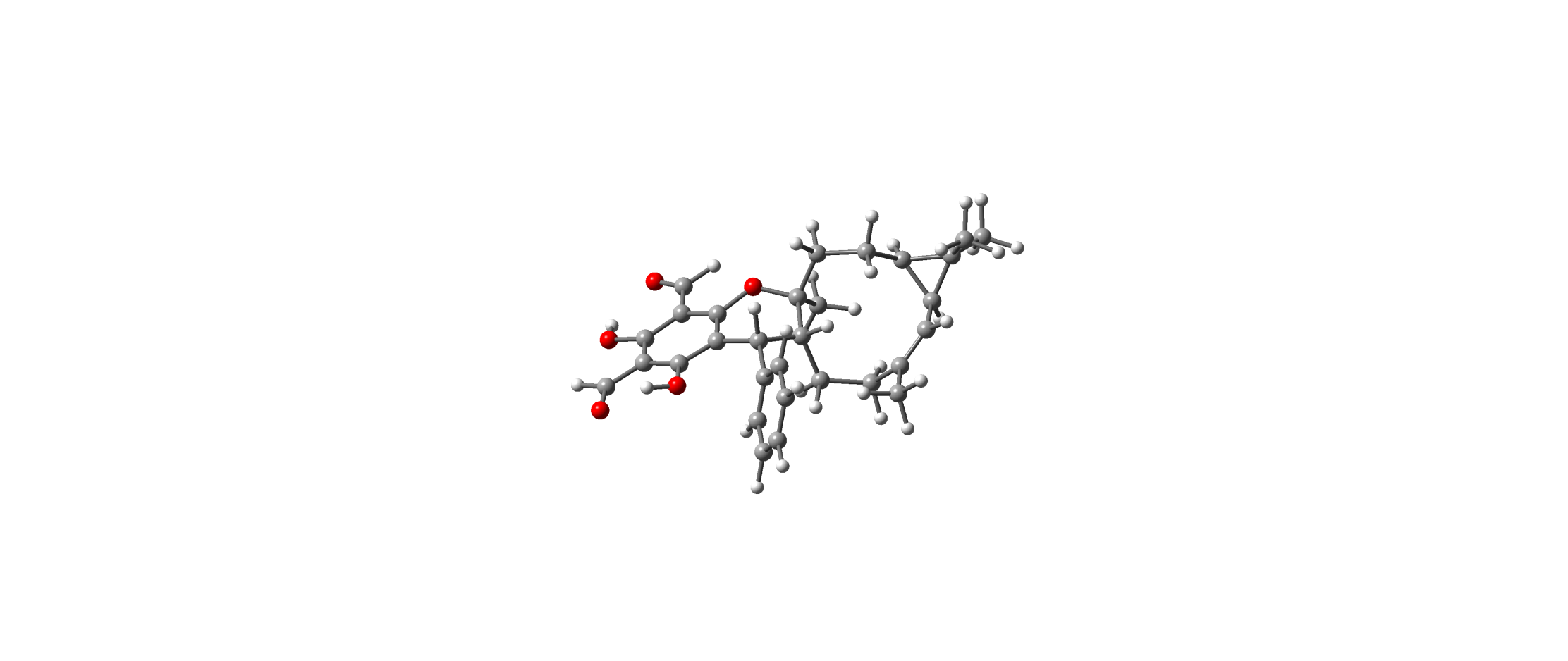


Standard orientation:

-------------------------------------------------------------------------------------------------------

Center Atomic Atomic Coordinates (Angstroms)

Number Number Type X Y Z

---------------------------------------------------------------------------------------------------------

1 6 0 3.460354 -2.286878 0.137992

2 6 0 3.617342 -1.407791 1.235318

3 6 0 2.725466 -0.298885 1.359871

4 6 0 1.695693 -0.076008 0.429588

5 6 0 1.554370 -0.971962 -0.618723

6 6 0 2.416634 -2.089149 -0.804841

7 6 0 0.834663 1.164675 0.440757

8 6 0 -0.646506 0.843612 0.006771

9 6 0 -0.761214 -0.330135 -1.042872

10 8 0 0.581907 -0.762517 -1.535036

11 6 0 -1.362238 2.179288 -0.321041

12 6 0 -2.907944 2.235608 -0.297371

13 6 0 -2.647689 -1.660986 0.378320

14 6 0 -1.328188 -1.638934 -0.419883

15 6 0 -3.573230 1.850032 1.006071

16 6 0 -4.409387 0.801743 1.098264

17 6 0 -4.782447 -0.113139 -0.022775

18 6 0 -3.923028 -1.335381 -0.384651

19 6 0 -5.298850 -1.518871 0.228188

20 6 0 -6.403053 -2.019835 -0.690438

21 6 0 -5.428491 -2.040114 1.650789

22 6 0 -3.296433 2.736061 2.196781

23 1 0 -1.111180 0.458543 0.920445

24 6 0 -1.449345 0.023038 -2.360488

25 8 0 4.301520 -3.313065 0.014137

26 8 0 2.864764 0.564867 2.365106

27 6 0 1.535973 2.295592 -0.325917

28 6 0 1.799110 2.233803 -1.703776

29 6 0 2.458481 3.277837 -2.352181

30 6 0 2.877111 4.402679 -1.637719

31 6 0 2.631991 4.471665 -0.267026

32 6 0 1.967573 3.426769 0.378868

33 6 0 2.249095 -3.016389 -1.898439

34 8 0 2.987463 -3.996944 -2.090292

35 6 0 4.670101 -1.602507 2.207654

36 8 0 4.856521 -0.853003 3.179327

37 1 0 0.764982 1.508723 1.476538

38 1 0 -0.994072 2.909766 0.409830

39 1 0 -1.022105 2.560364 -1.288964

40 1 0 -3.175091 3.279911 -0.522479

41 1 0 -3.325265 1.640001 -1.110536

42 1 0 -2.721049 -2.681503 0.780287

43 1 0 -2.561094 -1.006107 1.251838

44 1 0 -1.392094 -2.372502 -1.234270

45 1 0 -0.556923 -2.012847 0.265588

46 1 0 -4.874066 0.607352 2.065066

47 1 0 -5.237713 0.387680 -0.878264

48 1 0 -3.874846 -1.568648 -1.448312

49 1 0 -7.390660 -1.706963 -0.326974

50 1 0 -6.402257 -3.116220 -0.748242

51 1 0 -6.286158 -1.632865 -1.709214

52 1 0 -4.627678 -1.693631 2.309616

53 1 0 -6.382251 -1.724952 2.093384

54 1 0 -5.410688 -3.137484 1.659767

55 1 0 -2.229798 2.747781 2.458836

56 1 0 -3.574219 3.778696 1.987058

57 1 0 -3.852708 2.408807 3.080844

58 1 0 -1.005377 0.911002 -2.818734

59 1 0 -1.332825 -0.812604 -3.057524

60 1 0 -2.515350 0.197522 -2.217253

61 1 0 4.022659 -3.816618 -0.807920

62 1 0 3.642837 0.250069 2.914227

63 1 0 1.493236 1.361547 -2.272298

64 1 0 2.649436 3.209572 -3.420230

65 1 0 3.393055 5.213558 -2.144894

66 1 0 2.959373 5.336355 0.304502

67 1 0 1.793146 3.483655 1.450581

68 1 0 1.413570 -2.825330 -2.588728

69 1 0 5.330288 -2.469961 2.057941

-----------------------------------------------------------------------------------------------------

Optimized structure of 4*R*,5*S*,6*S*,7*R*,1*'S*-**3**


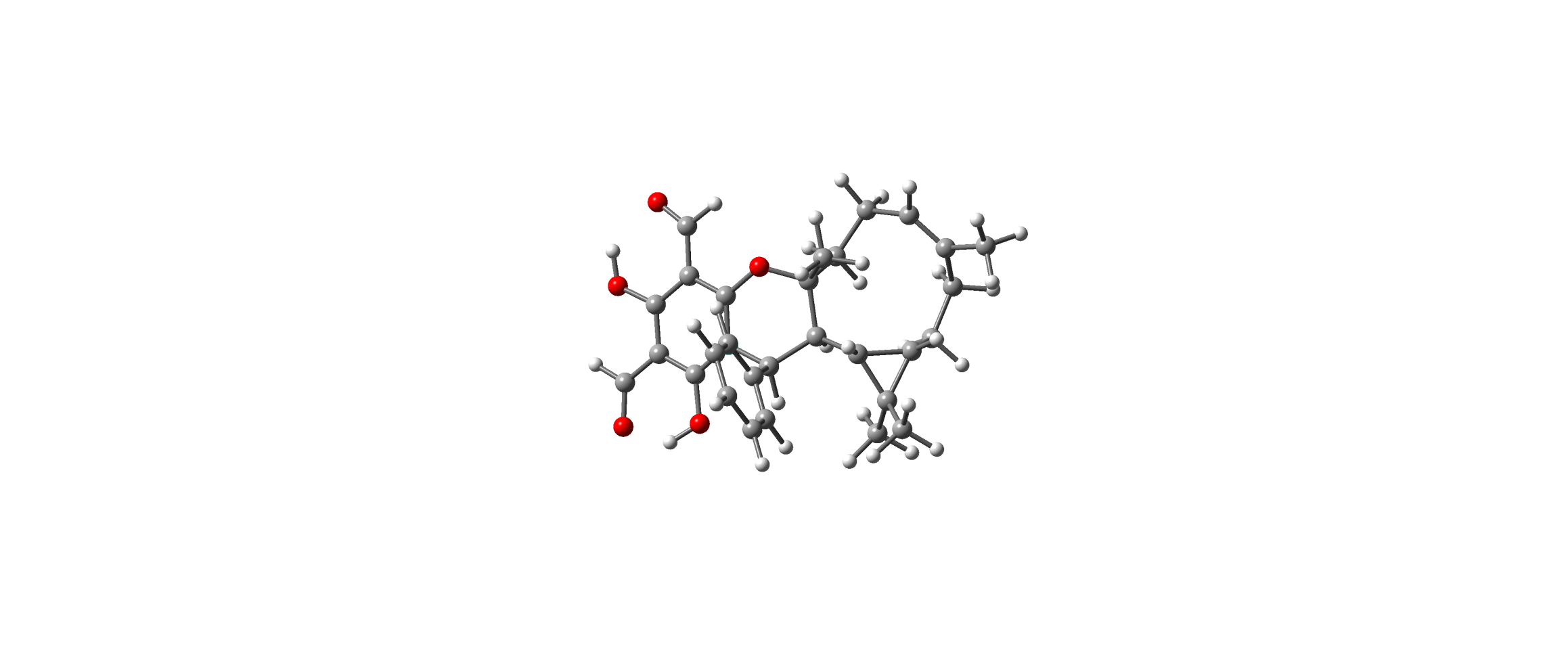


Standard orientation:

------------------------------------------------------------------------------------------------------------------

Center Atomic Atomic Coordinates (Angstroms)

Number Number Type X Y Z

------------------------------------------------------------------------------------------------------------------

1 6 0 1.658316 -1.000632 0.471040

2 6 0 1.590210 0.101227 -0.368528

3 6 0 2.712947 0.406883 -1.156155

4 6 0 3.905784 -0.377399 -1.093759

5 6 0 3.949268 -1.479227 -0.207415

6 6 0 2.820170 -1.816144 0.585477

7 8 0 0.604722 -1.293636 1.263826

8 6 0 -0.777587 -1.179494 0.713143

9 6 0 -0.932200 0.073207 -0.241813

10 6 0 0.356597 0.972528 -0.317136

11 6 0 -0.941983 -2.531779 -0.030042

12 6 0 -2.248188 -2.850915 -0.790692

13 6 0 -3.549066 0.797041 -0.598419

14 6 0 -2.169236 0.926308 0.048540

15 6 0 -3.503769 -2.838547 0.037740

16 6 0 -4.642782 -2.164298 -0.181605

17 6 0 -4.930633 -1.250061 -1.368884

18 6 0 -3.927038 -0.134411 -1.740671

19 6 0 -1.617254 -1.124260 1.988760

20 6 0 0.506898 2.072788 0.741962

21 6 0 0.580134 3.407757 0.321000

22 6 0 0.748641 4.449741 1.235351

23 6 0 0.851818 4.173810 2.598401

24 6 0 0.799329 2.847899 3.032044

25 6 0 0.636435 1.809113 2.114864

26 6 0 -5.812579 -2.334453 0.762214

27 6 0 -2.700147 2.029129 -0.859415

28 6 0 -2.149945 2.248605 -2.262609

29 6 0 -3.131143 3.326880 -0.190866

30 8 0 5.065974 -2.203431 -0.140297

31 8 0 2.648424 1.465307 -1.963892

32 1 0 -1.016199 -0.339148 -1.253657

33 6 0 2.874137 -2.959691 1.464587

34 8 0 3.873294 -3.687771 1.589013

35 6 0 5.045460 -0.027837 -1.912004

36 8 0 5.060149 0.932992 -2.697942

37 1 0 0.286861 1.492928 -1.274093

38 1 0 -0.768904 -3.317802 0.716036

39 1 0 -0.118241 -2.606740 -0.752590

40 1 0 -2.110499 -3.866454 -1.193798

41 1 0 -2.344162 -2.205711 -1.664236

42 1 0 -4.375558 0.958760 0.095742

43 1 0 -2.221062 1.212740 1.095503

44 1 0 -3.467235 -3.483742 0.917908

45 1 0 -5.095485 -1.868661 -2.263872

46 1 0 -5.895652 -0.761792 -1.179820

47 1 0 -3.035300 -0.552749 -2.213145

48 1 0 -4.410423 0.460976 -2.528707

49 1 0 -2.680555 -1.115156 1.748531

50 1 0 -1.405104 -2.007449 2.599659

51 1 0 -1.379833 -0.237461 2.581858

52 1 0 0.521716 3.633141 -0.740649

53 1 0 0.805323 5.475014 0.878654

54 1 0 0.981600 4.981028 3.314277

55 1 0 0.893767 2.617191 4.090209

56 1 0 0.627804 0.784940 2.470471

57 1 0 -5.590610 -3.034790 1.573655

58 1 0 -6.102849 -1.373476 1.210032

59 1 0 -6.699089 -2.705631 0.228577

60 1 0 -1.350058 2.998195 -2.254734

61 1 0 -2.938478 2.628171 -2.925975

62 1 0 -1.748489 1.340840 -2.722072

63 1 0 -3.572822 3.141588 0.794943

64 1 0 -2.280078 4.003730 -0.050841

65 1 0 -3.882427 3.848173 -0.799430

66 1 0 4.896359 -2.933320 0.527429

67 1 0 3.532277 1.526996 -2.434262

68 1 0 1.968304 -3.181776 2.048744

69 1 0 5.939040 -0.662844 -1.816679

-------------------------------------------------------------------------------------------------------------

**Original NMR, IR and CD spectra**


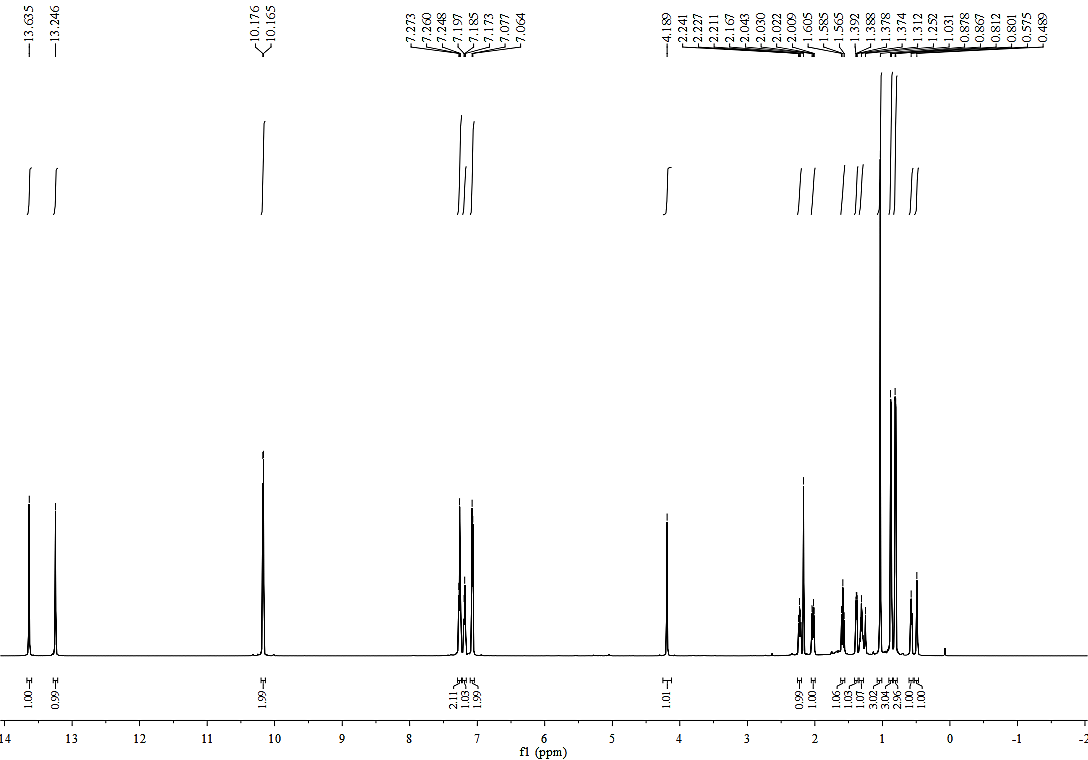


**Figure S1.** ^1^H NMR spectrum of guajavadial A (**1**) (600 MHz, CDCl_3_)


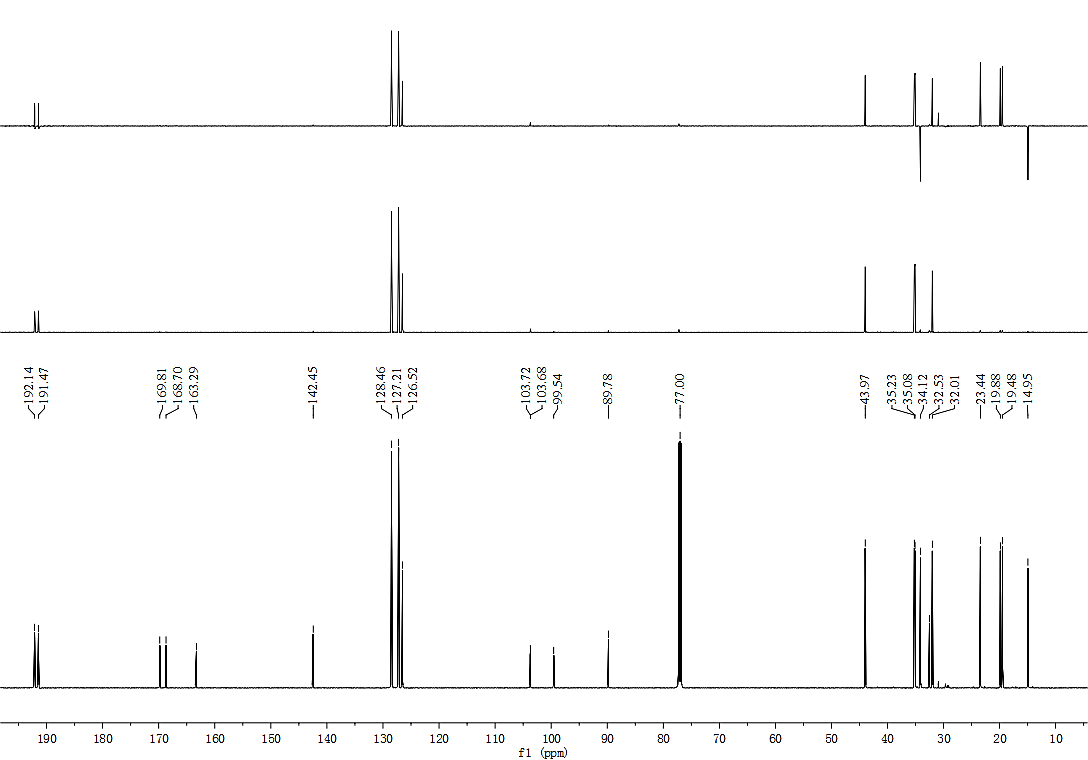


**Figure S2.** ^13^C NMR spectrum of guajavadial A (**1**) (150 MHz, CDCl_3_)


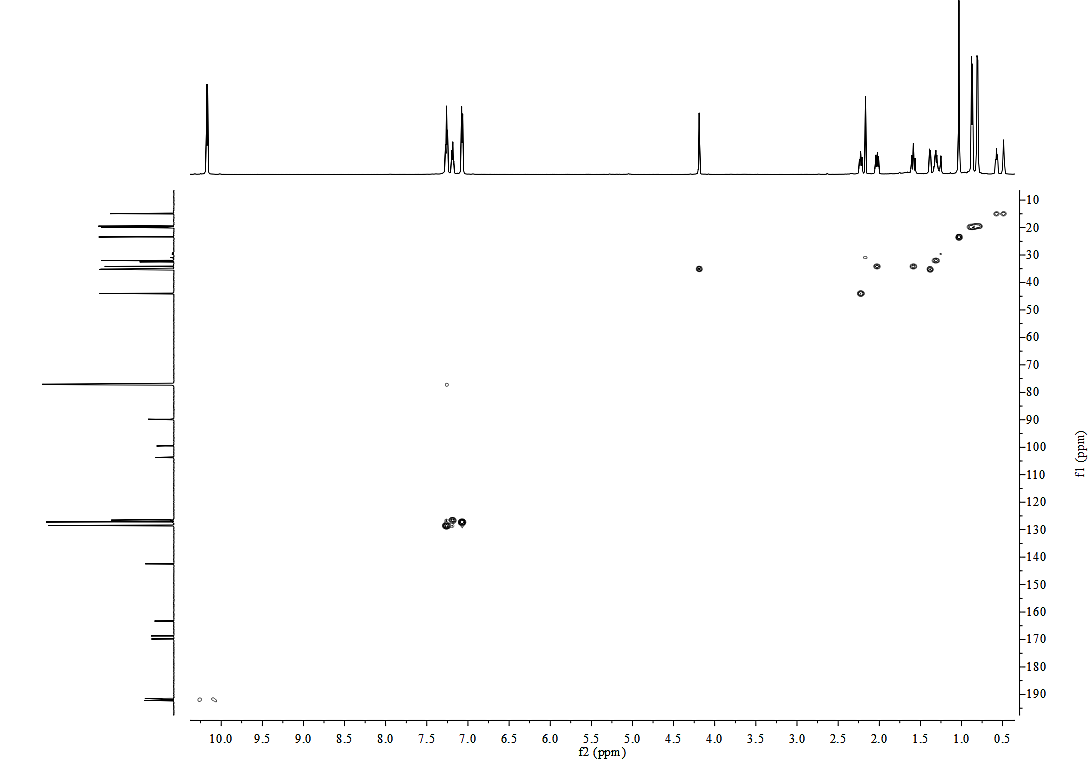


**Figure S3.** HSQC spectrum of guajavadial A (**1**) (600 MHz, CDCl_3_)


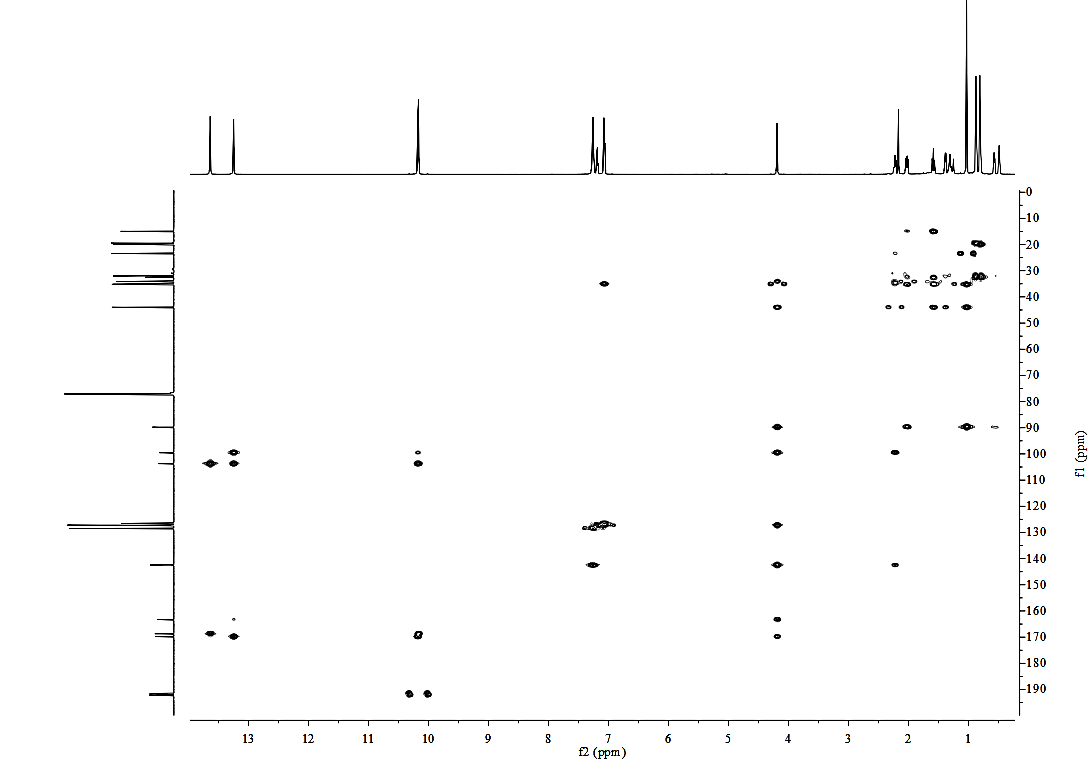


**Figure S4.** HMBC spectrum of guajavadial A (**1**) (600 MHz, CDCl_3_)


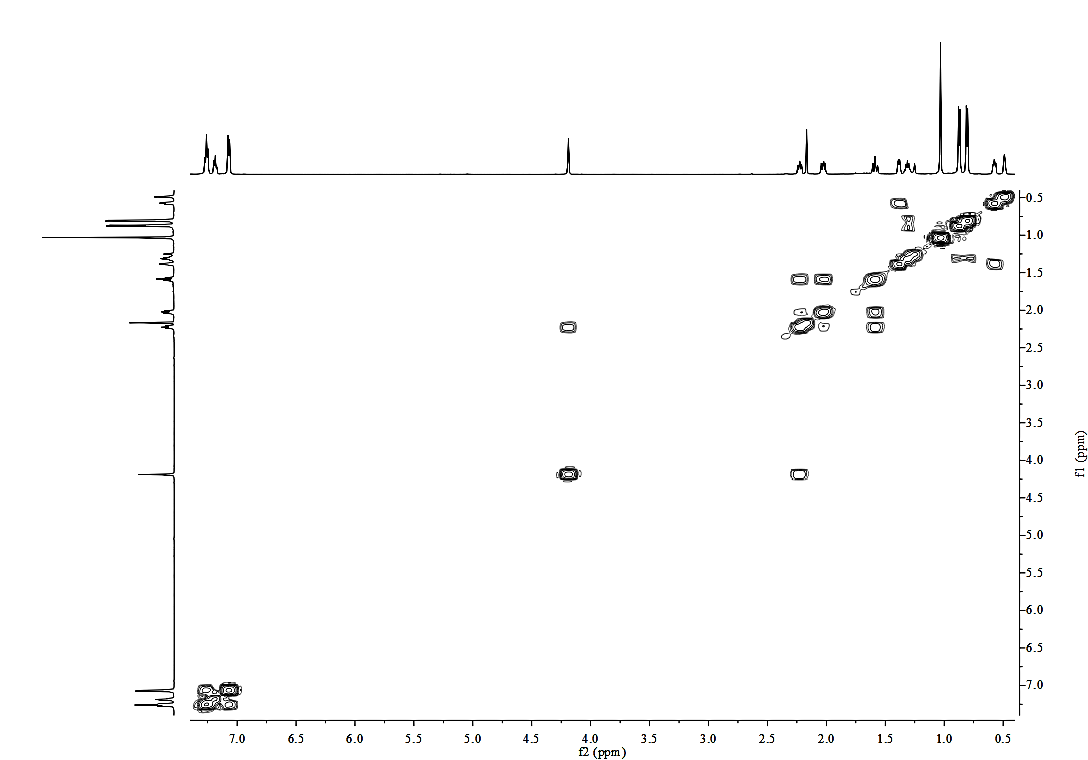


**Figure S5.** ^1^H–^1^H COSY spectrum of guajavadial A (**1**) (600 MHz, CDCl_3_)


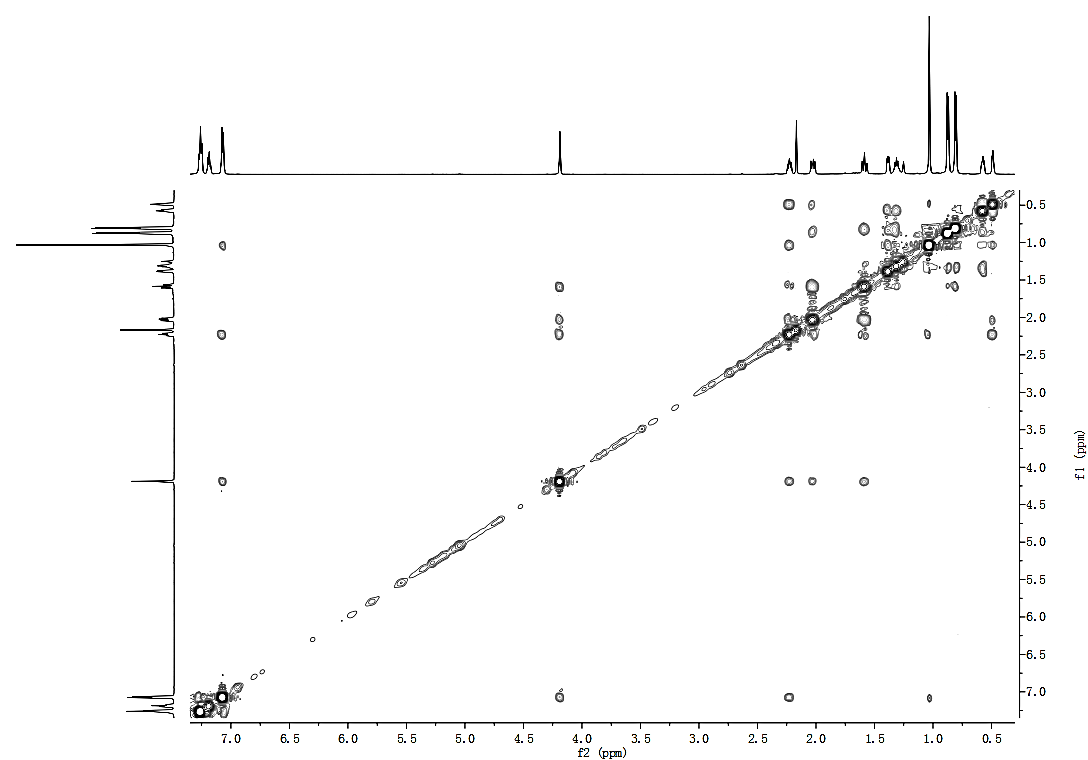


**Figure S6.** NOESY spectrum of guajavadial A (**1**) (600 MHz, CDCl_3_)

**Figure S7.** HRESIMS spectrum of guajavadial A (**1**)


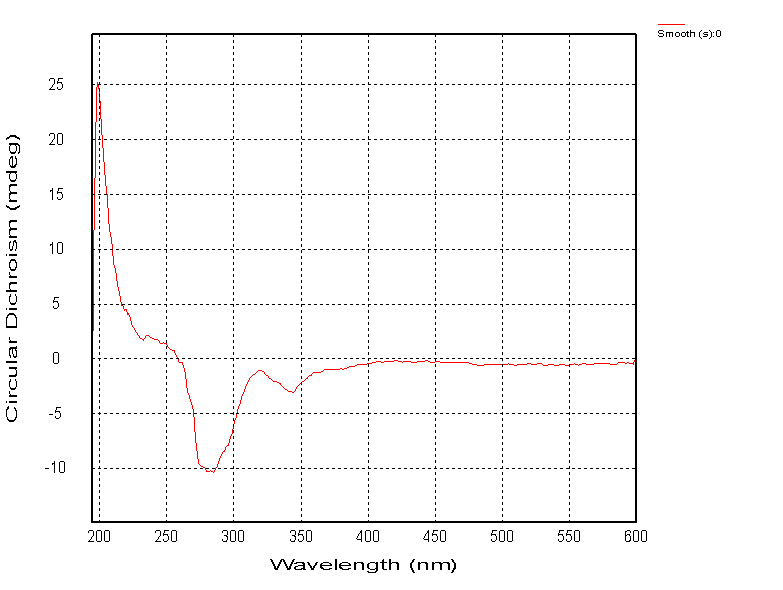


**Figure S8.** CD spectrum of guajavadial A (**1**)


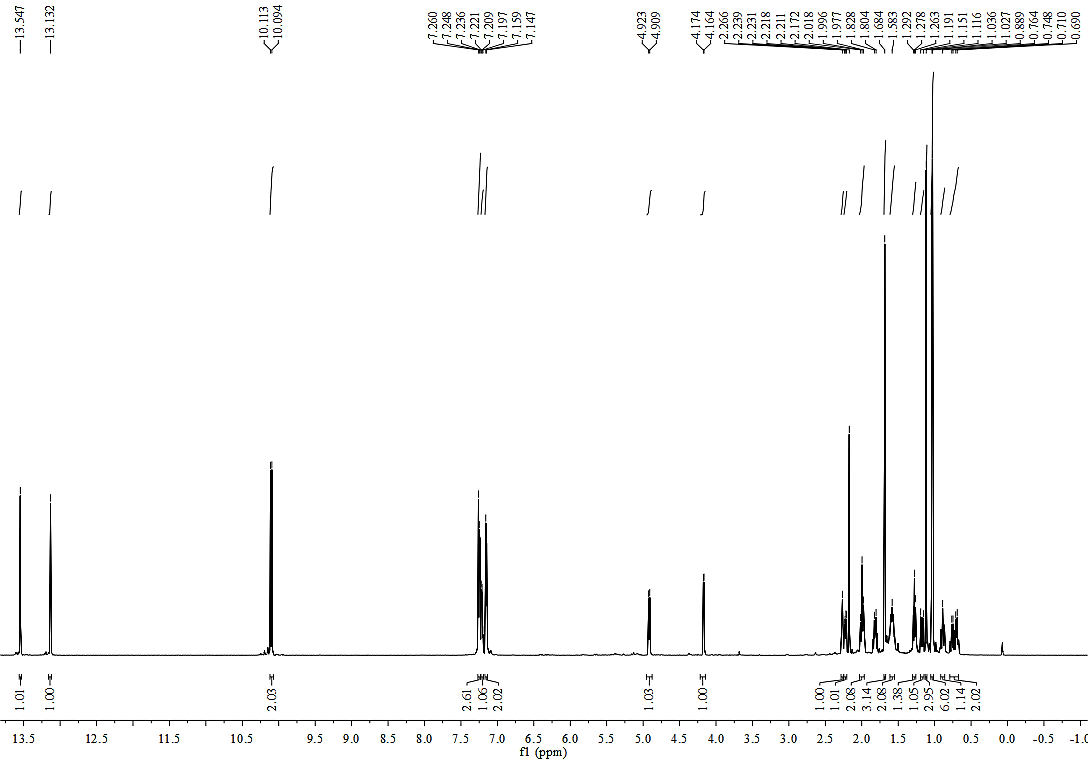


**Figure S9.** ^1^H NMR spectrum of guajavadial B (**2**) (600 MHz, CDCl_3_)


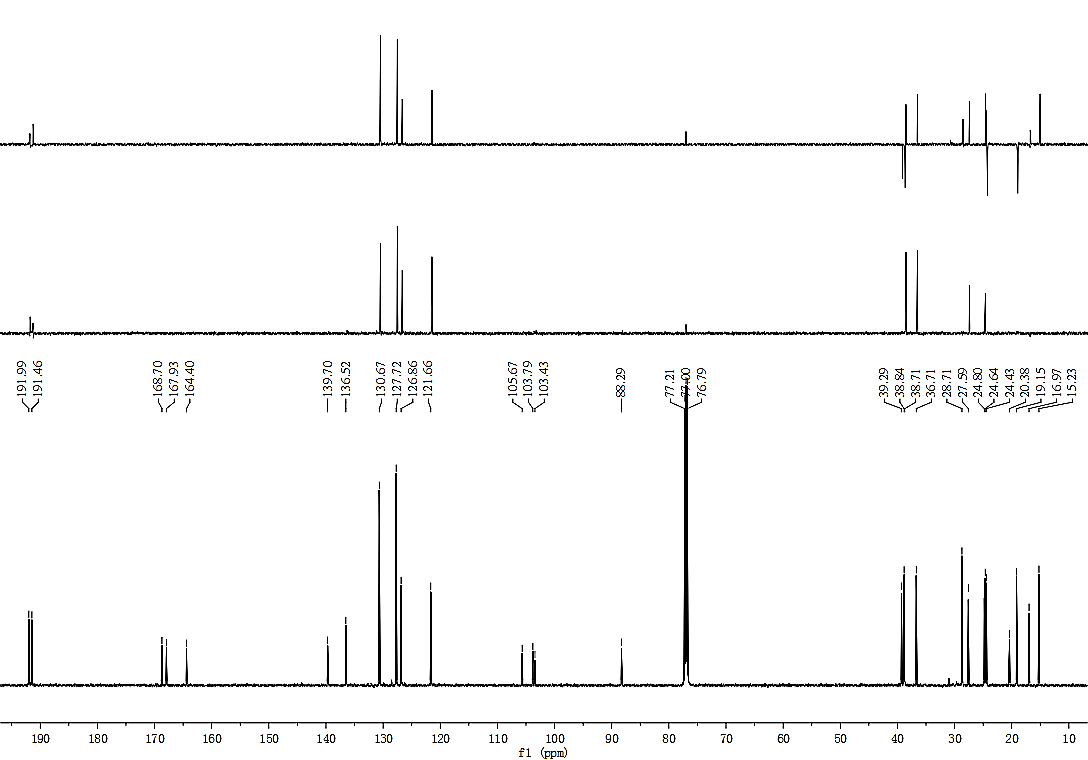


**Figure S10.** ^13^C NMR spectrum of guajavadial B (**2**) (150 MHz, CDCl_3_)


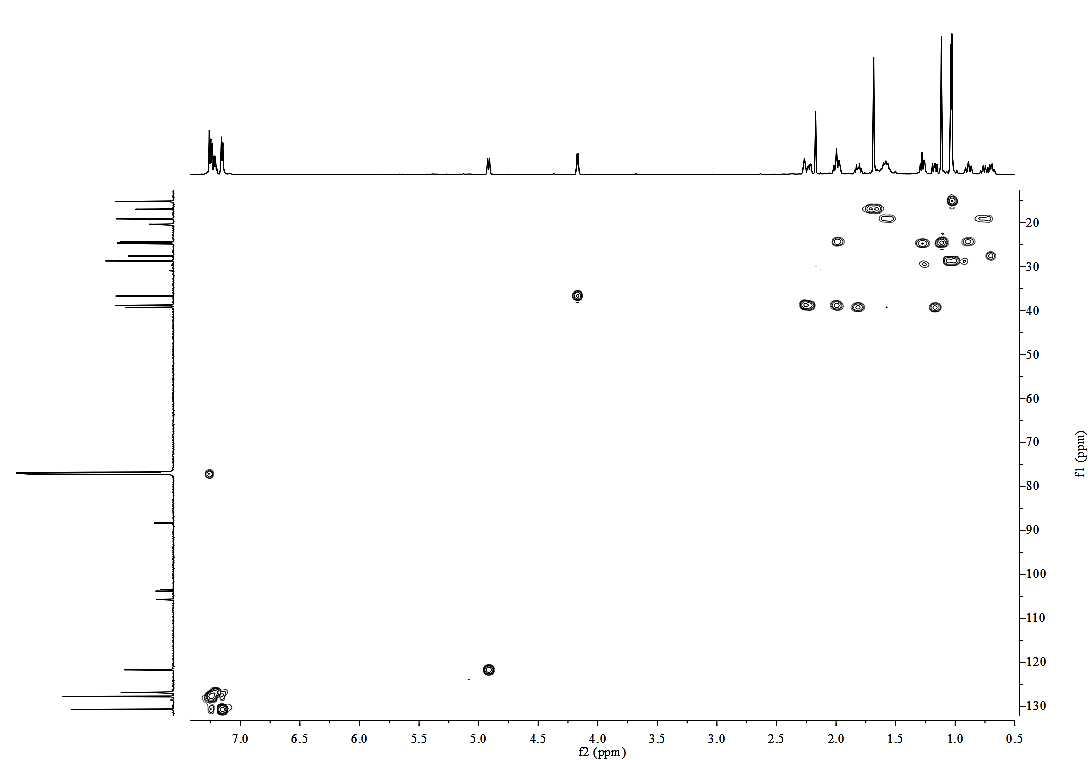


**Figure S11.** HSQC spectrum of guajavadial B (**2**) (600 MHz, CDCl_3_)

**
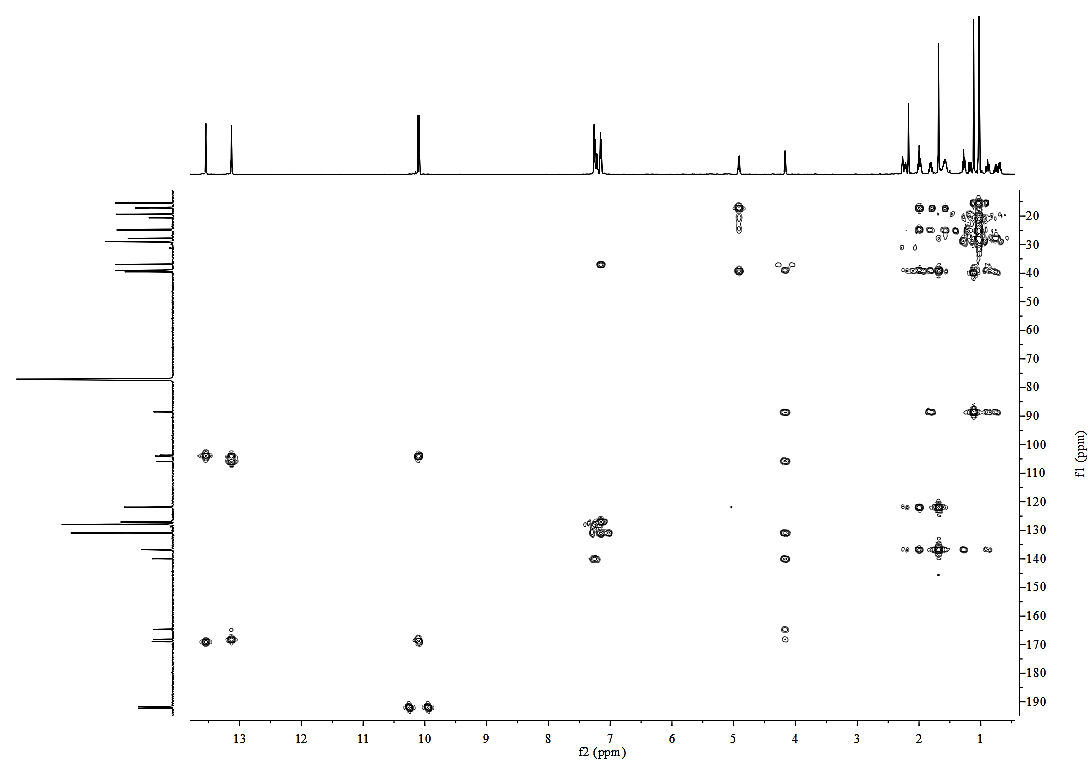
**

**Figure S12.** HMBC spectrum of guajavadial B (**2**) (600 MHz, CDCl_3_)


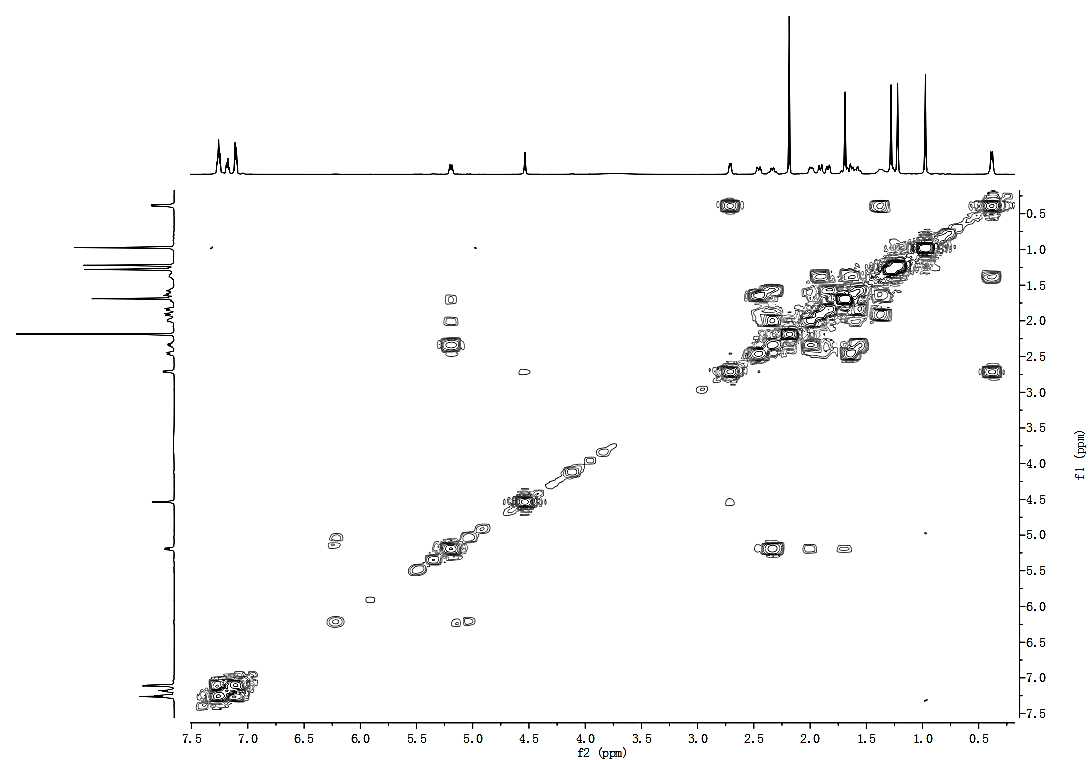


**Figure S13.** ^1^H–^1^H COSY spectrum of guajavadial B (**2**) (600 MHz, CDCl_3_)

**
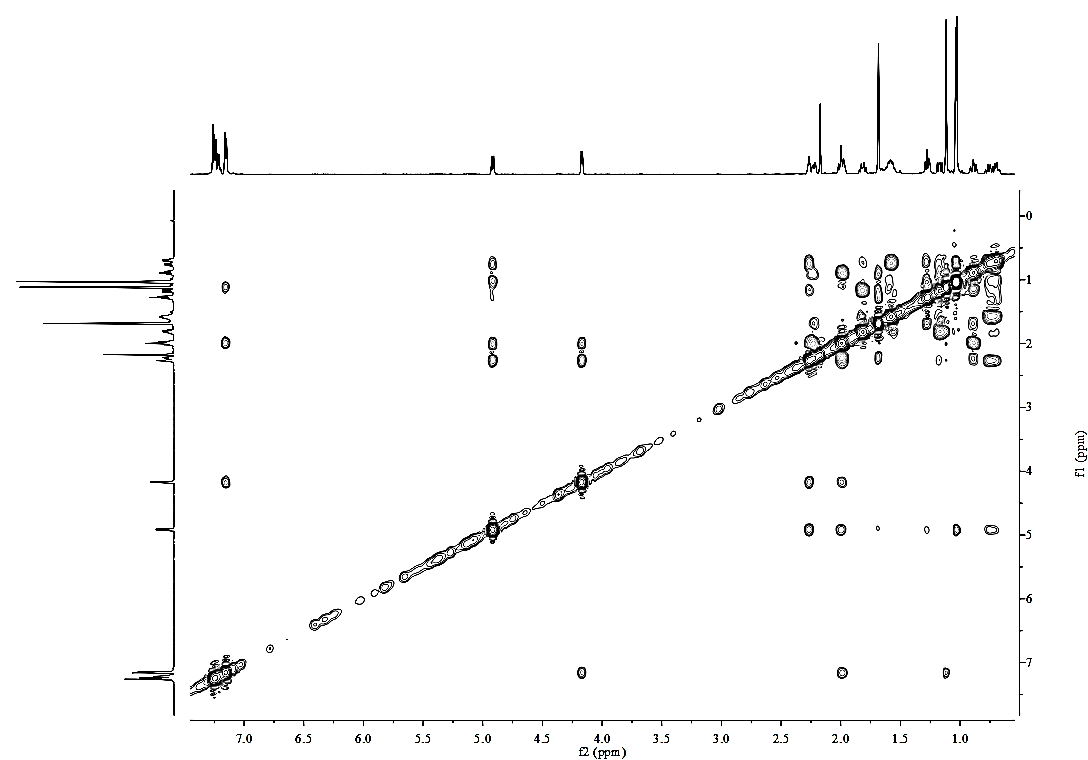
**

**Figure S14.** NOESY spectrum of guajavadial B (**2**) (600 MHz, CDCl_3_)

**Figure S15.** HRESIMS spectrum of guajavadial B (**2**)


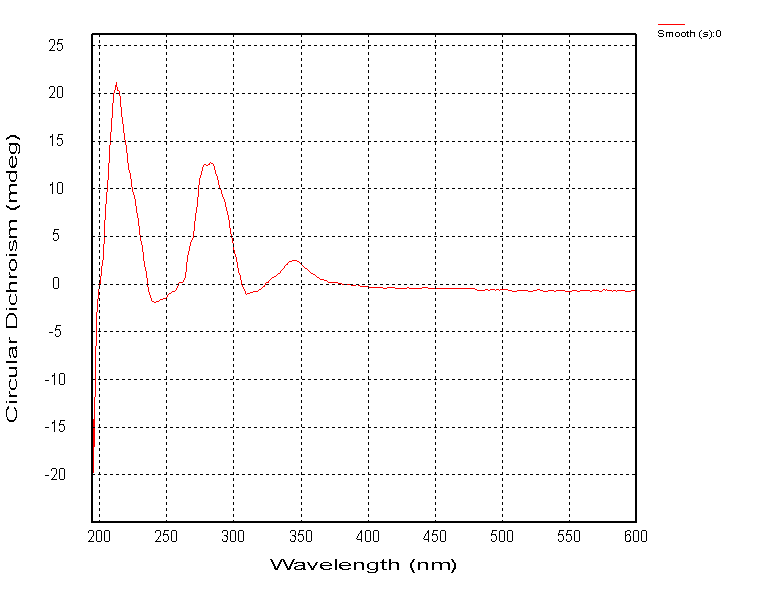


**Figure S16.** CD spectrum of guajavadial B (**2**)


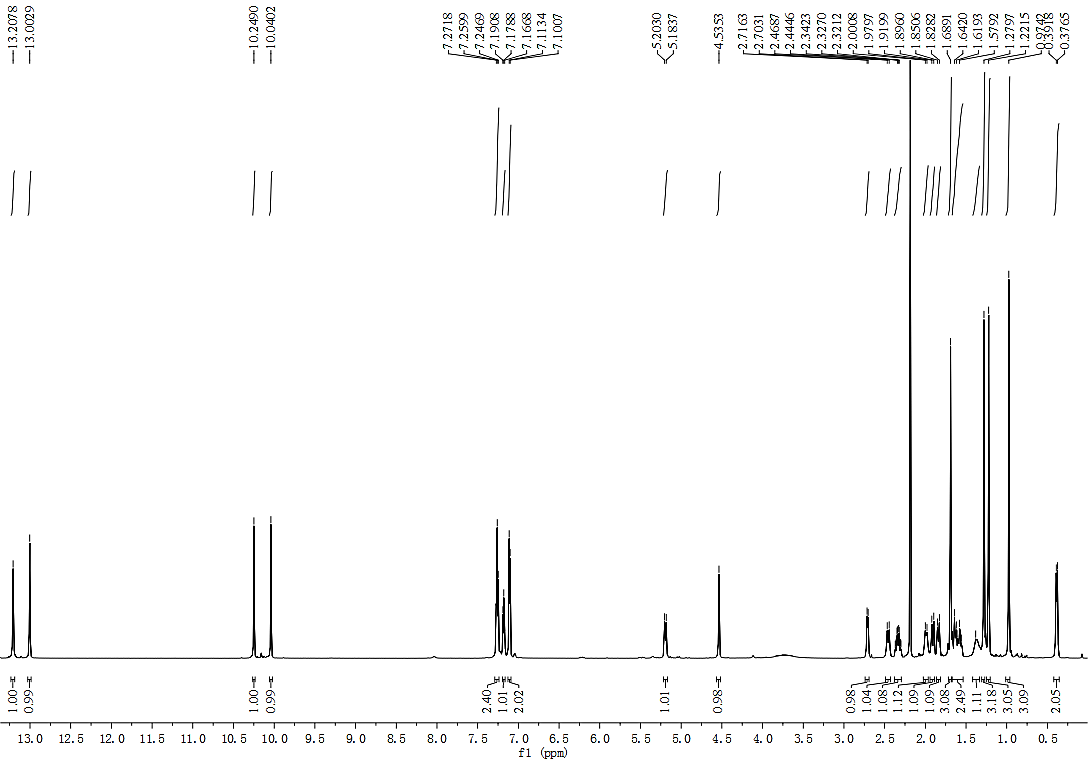


**Figure S17.** ^1^H NMR spectrum of guajavadial C (**3**) (600 MHz, CDCl_3_)


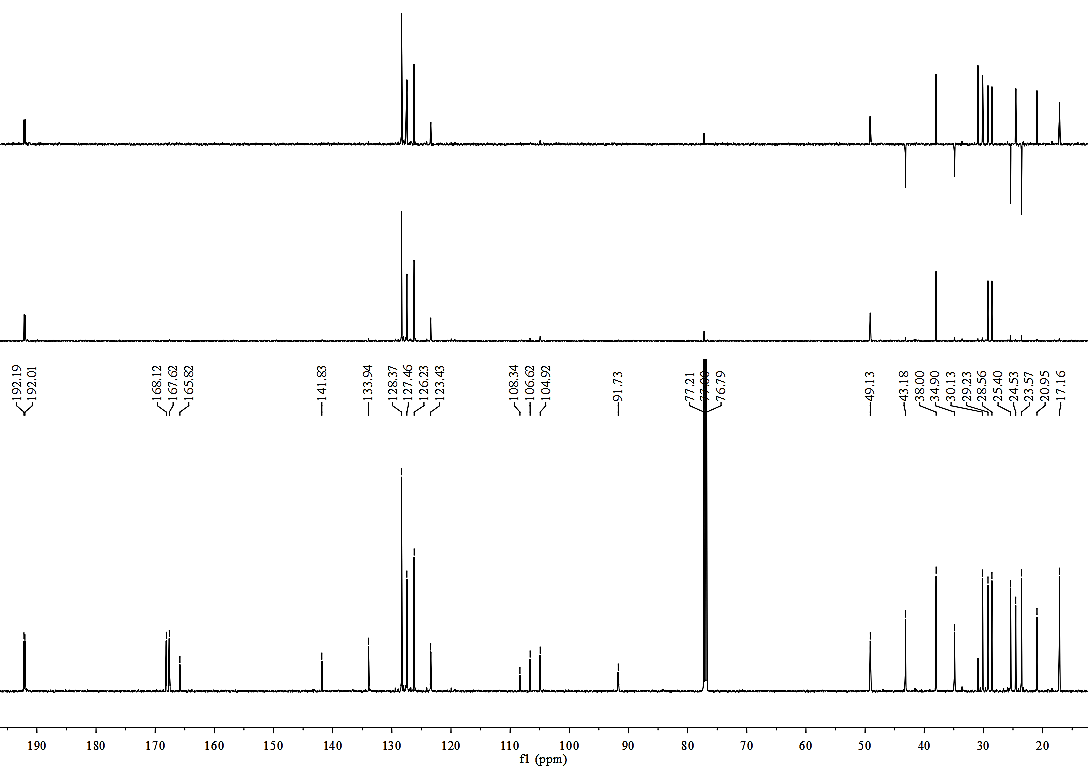


**Figure S18.** ^13^C NMR spectrum of guajavadial C (**3**) (150 MHz, CDCl_3_)


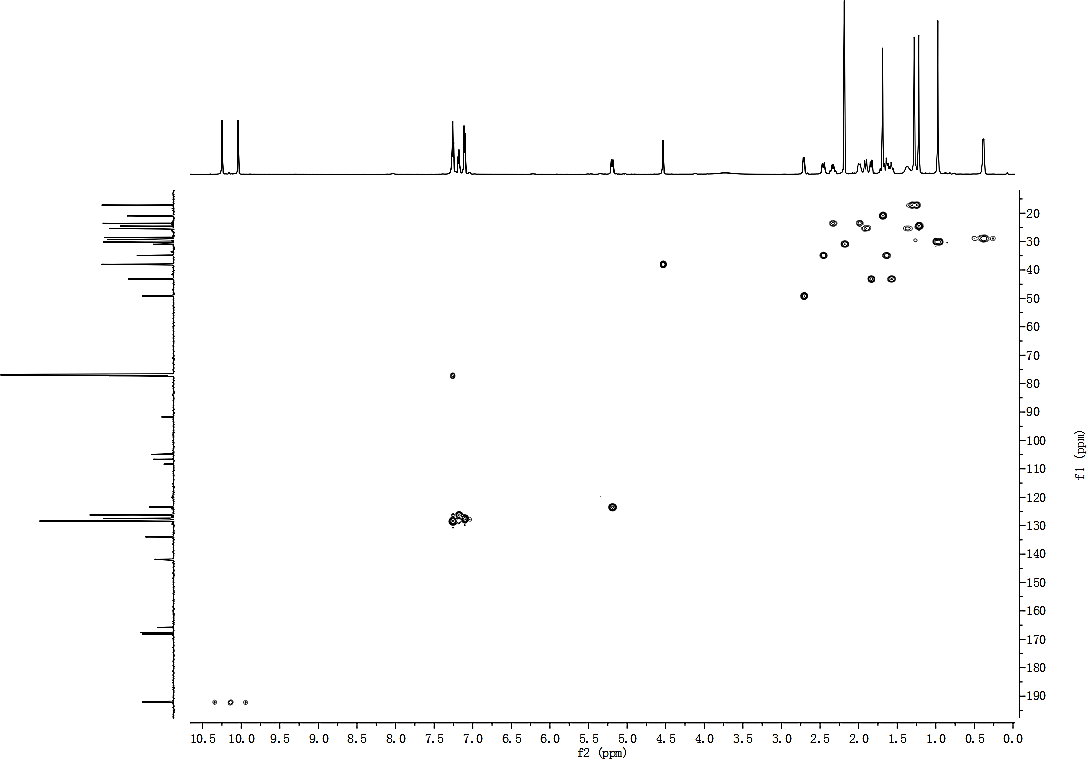


**Figure S19.** HSQC spectrum of guajavadial C (**3**) (600 MHz, CDCl_3_)


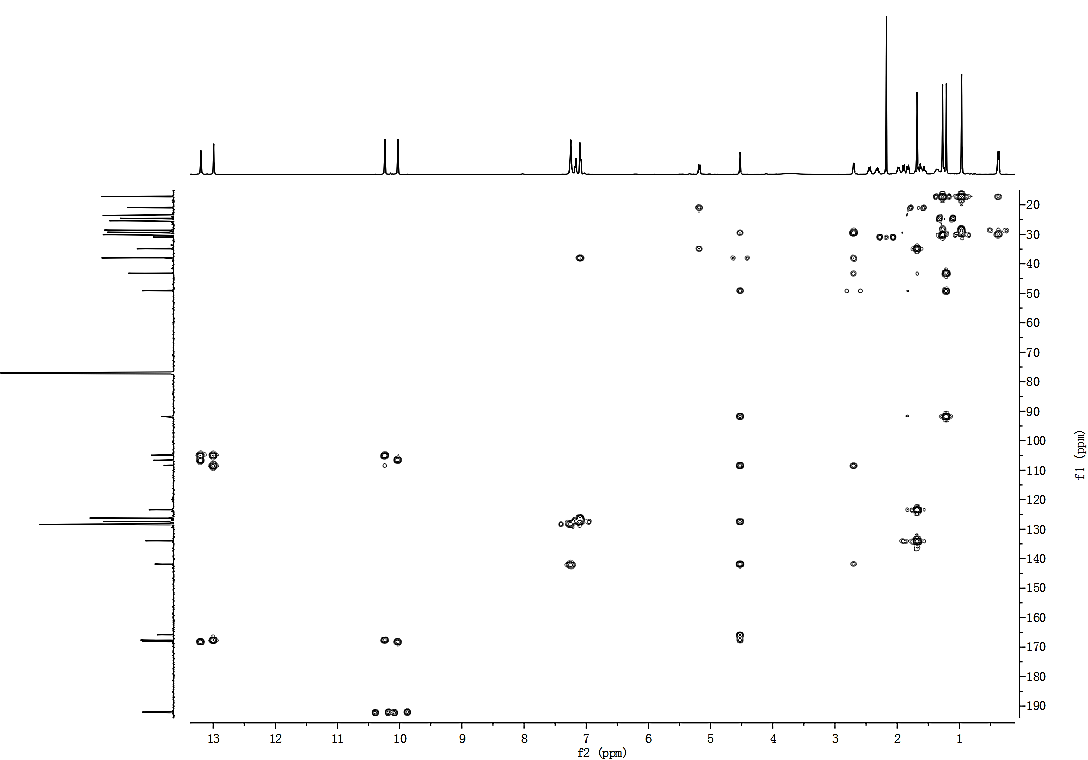


**Figure S20.** HMBC spectrum of guajavadial C (**3**) (600 MHz, CDCl_3_)


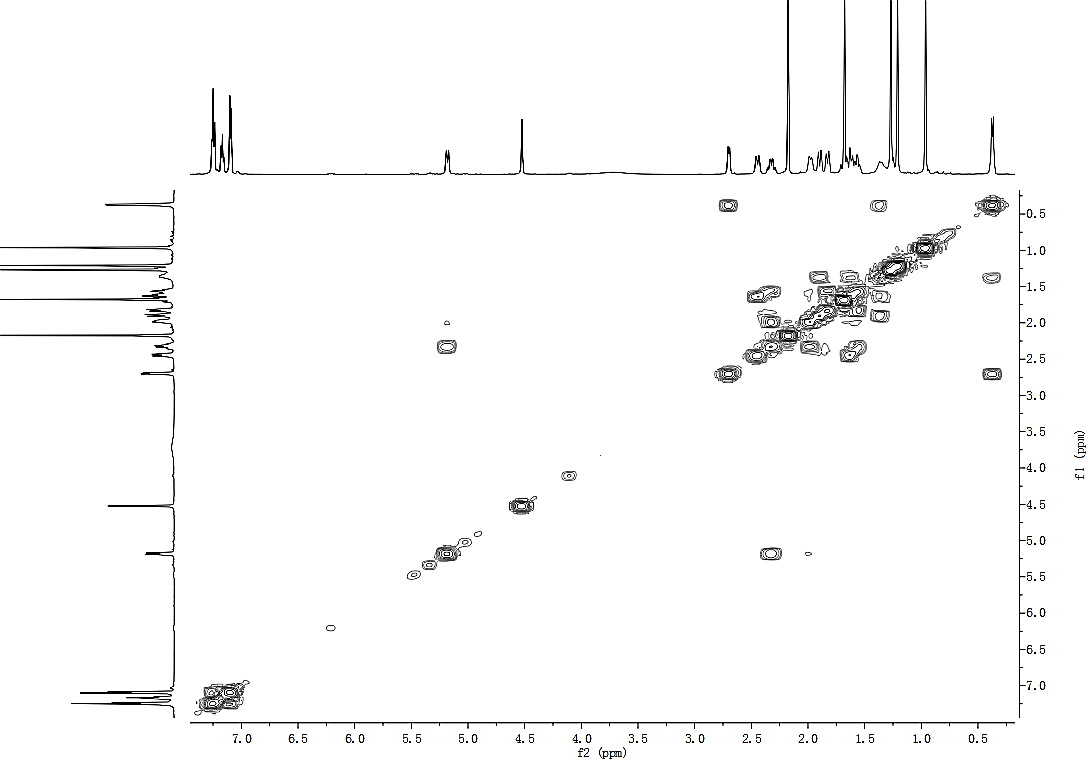


**Figure S21.** ^1^H–^1^H COSY spectrum of guajavadial C (**3**) (600 MHz, CDCl_3_)


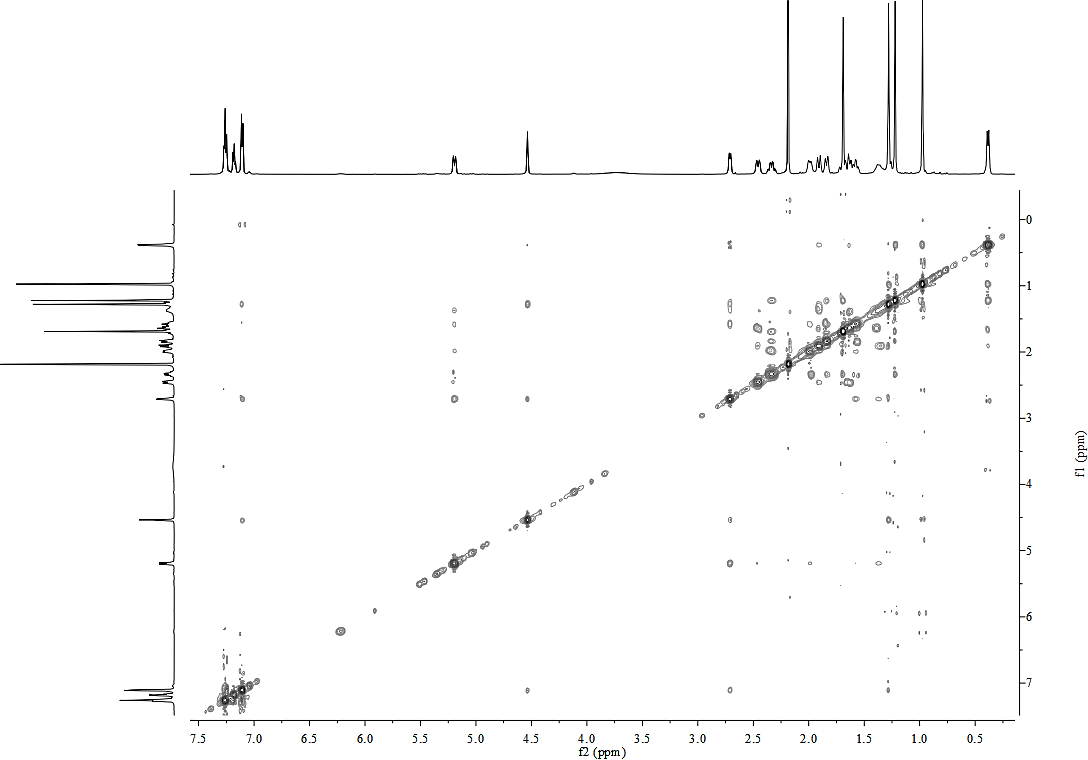


**Figure S22.** NOESY spectrum of guajavadial C (**3**) (600 MHz, CDCl_3_)

**Figure S23.** HRESIMS spectrum of guajavadial C (**3**)


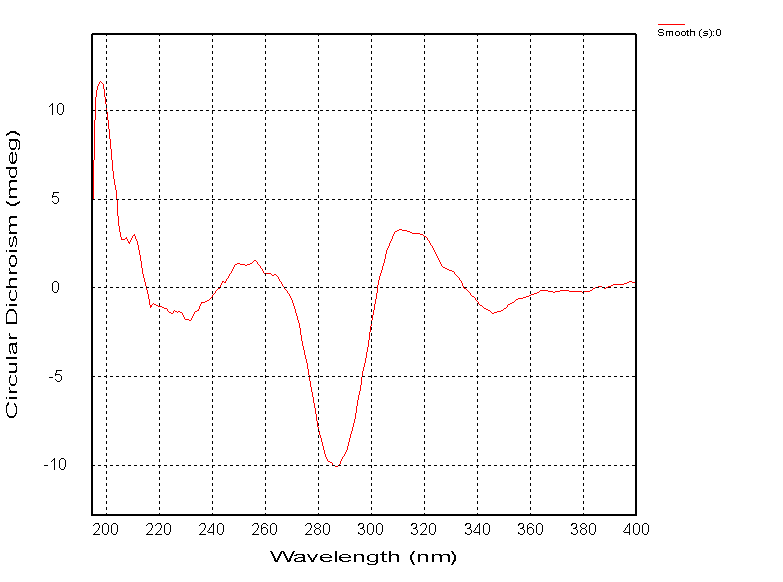


**Figure S24.** CD spectrum of guajavadial C (**3**)
